# Bacteria-specific pro-photosensitizer kills multidrug-resistant *Staphylococcus aureus* and *Pseudomonas aeruginosa*

**DOI:** 10.1038/s42003-021-01956-y

**Published:** 2021-03-25

**Authors:** Min Lu, Yongli Li, Mei X. Wu

**Affiliations:** 1 Department of Orthopaedics, Shanghai Key Laboratory for Prevention and Treatment of Bone and Joint Diseases, Shanghai Institute of Traumatology and Orthopaedics, Ruijin Hospital, Shanghai Jiao Tong University School of Medicine, Shanghai, People’s Republic of China; 2 Wellman Center for Photomedicine, Massachusetts General Hospital, Department of Dermatology, Harvard Medical School, Boston, MA USA

**Keywords:** Antimicrobial resistance, Clinical microbiology

## Abstract

The emergence of multidrug-resistant bacteria has become a real threat and we are fast running out of treatment options. A combinatory strategy is explored here to eradicate multidrug-resistant *Staphlococcus aureus* and *Pseudomonas aeruginosa* including planktonic cells, established biofilms, and persisters as high as 7.5 log bacteria in less than 30 min. Blue-laser and thymol together rapidly sterilized acute infected or biofilm-associated wounds and successfully prevented systematic dissemination in mice. Mechanistically, blue-laser and thymol instigated oxidative bursts exclusively in bacteria owing to abundant proporphyrin-like compounds produced in bacteria over mammalian cells, which transformed harmless thymol into blue-laser sensitizers, thymoquinone and thymohydroquinone. Photo-excitations of thymoquinone and thymohydroquinone augmented reactive oxygen species production and initiated a torrent of cytotoxic events in bacteria while completely sparing the host tissue. The investigation unravels a previously unappreciated property of thymol as a pro-photosensitizer analogous to a prodrug that is activated only in bacteria.

## Abstract (web summary)

Multidrug-resistant bacteria are a real threat to human health. Here, the authors investigate a combinatory strategy using blue-laser and thymol against *Staphylococcus aureus* and *Pseudomonas aeruginosa*. Blue-laser and thymol succesfully sterilized acute infected or biofilm-associated wounds and prevented systematic dissemination in mice. Compared with mammalian cells, bacteria contain abundant proporphyrin-like compounds that transform harmless thymol into blue-laser sensitizers, thymoquinone and thymohydroquinone. Photo-excitation of thymoquinone and thymohydroquinone augmented reactive oxygen species production in bacteria while completely sparing the host tissue.

## Introduction

Multidrug-resistant (MDR) superbugs have been emerging as a real threat rather than a looming crisis^1^. In particular, the Gram-positive methicillin-resistant *Staphylococcus aureus* (MRSA) and Gram-negative *Pseudomonas aeruginosa* (*Pa*) are listed by WHO as prioritized nosocomial pathogens, which require much research and development of new antimicrobial therapeutics^2^. MRSA and *Pa* are strong biofilm producers; generating biofilms with a poor permeability and a slow metabolic rate of the encased bacteria, further challenging antibiotic treatments^3^. Moreover, dormant persisters spontaneously form in the biofilm population. The recalcitrant persisters can dodge antibiotic attacks and lead to recurrent or chronic infections^4^. The unmet need for anti-biofilm and anti-persister therapeutics has urged researches on non-antibiotic alternatives^5^.

MRSA and *Pa* are the top two MDR species frequently found at open skin wounds in association with sepsis, a lethal disease, if the infection is left unchecked. Open wound beds are desirable for biofilm establishment and provide portals for bacterial invasion and systematic dissemination. Treatment options are limited should MDR bacteria contaminate the wounds^6^. The nephrotoxic and neurotoxic colistin appears to be the last resort for treating MDR biofilms at burns^7^. Essential oils from aromatic plants have been used as folkloric medicines for wound healing and their antimicrobial effect are appreciated throughout history and around the world. In nature, essential oils protect plants from phytopathogens and reduce bacterial burdens of MDR pathogens in trauma-associated wounds by targeting the membrane. As mutations in membrane synthesis likely affect bacterial fitness, resistance to essential oils hardly developed^8^. Blue light (BL) at 400–495 nm is another rising antimicrobial approach, particularly for skin infection. Many studies have validated BL’s efficacy in killing bacteria, regardless of their resistance profiles^9–11^. The exact mechanism of action for BL remains unclear. However, it is generally accepted that BL excites endogenous proporphyrin-like derivatives and stimulates a chain of reactive oxygen species (ROS) production. The toxic ROS react with multiple components of a bacterium and eventually rupture the cell. The nonselective and fast-acting characters of ROS minimize a chance for bacterial resistance to BL.

We herein report an antimicrobial synergy between BL and thymol. Thymol is a phenolic monoterpenoid that is commonly present in edible essential oils. The combined thymol and BL readily and safely inactivated all forms of bacteria, including planktonic cells, mature biofilms, and persisters of MDR MRSA and *Pa* strains in vivo and in vitro. In the combinatory therapy, thymol acted as a “pro-photosensitizer” and was oxidized to thymoquinone (TQ) and thymohydroquinone (THQ) exclusively in bacteria by BL. The resultant TQ and THQ acted as photosensitizers and magnified ROS productions exponentially and rapidly killed the pathogens while completely sparing the host tissue.

## Results

### Screening bactericidal activity of BL combined with different constituents of essential oils

**Fig. 1:**
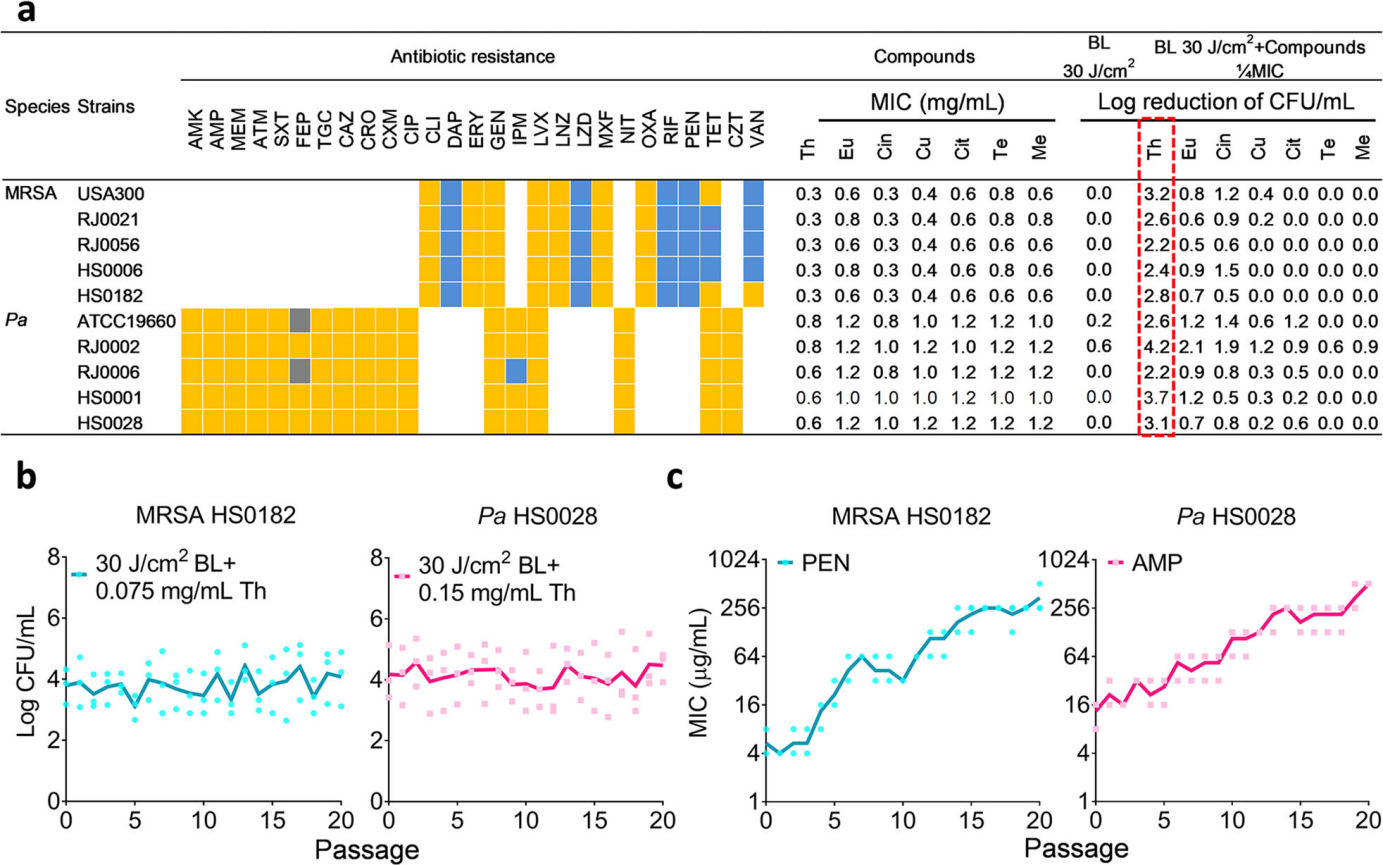
Screening bactericidal activity of BL combined with essential oil compounds. **a** Bacteria of 5 MRSA and 5 *Pa* strains were tested for their susceptibility to various antibiotics: susceptible (blue boxes), intermediate (gray boxes), or resistant (yellow boxes) and MIC values to Th (thymol), Eu (eugenol), Cin (cinnamaldehyde), Cu (cuminaldehyde), Cit (citral-a), Te (terpinen-4-ol), and Me (menthol). Bactericidal efficacies of 30 J/cm^2^ BL in the absence and presence of an indicated compound at 1/4 MICs were obtained against the 10 MDR strains. **b** and **c** Resistance development of MRSA HS0182 and *Pa* HS0028 to the sub-lethal doses of BL plus thymol (**b**), penicillin (PEN) alone (**c**, left), or ampicillin (AMP) alone (**c**, right), respectively. Each value represents the mean of triplicate assays. Antibiotics in **a**: AMK, amikacin; MEM, meropenem; ATM, aztreonam; SXT, trimethoprim-sulfamethoxazole; FEP, cefepime; TGC, tigecycline; CAZ, ceftazidime; CRO, ceftriaxone; CXM, cefuroxime; CIP, ciprofloxacin; CLI, clindamycin; DAP, daptomycin; ERY, erythromycin; GEN, gentamicin; IPM, imipenem; LVX, levofloxacin; LNZ, linezolid; LZD, linezolid; MXF, moxifloxacin; NIT, nitrofurantoin; OXA, oxacillin; RIF, rifampin; TET, tetracycline; CZT, ceftizoxime; and VAN, vancomycin.

All bacterial strains used in this study, including four MRSA clinical isolates, four *Pa* clinical isolates, one luminescent strain of USA300, and one standard strain of *Pa* ATCC19660, were confirmed MDR by microbiological tests (Fig. 1a and Supplementary Table 1). Two dozen of compounds were screened and seven of them were shown in Fig. 1a in which thymol exhibited the most potent bactericidal activities against the MDR strains with a minimal inhibitory concentration (MIC) range from 0.3 mg/mL to 0.8 mg/mL dependent on the bacterial strain tested. BL alone at 30 J/cm^2^ was ineffective in killing the MDR strains; only marginal reductions of 0.2- and 0.6-log CFU/mL were observed for *Pa* ATCC19660 and *Pa* RJ0002, respectively. Combinations of BL (30 J/cm^2^) with each of the seven compounds at 1/4 MIC enhanced the antimicrobial activity in 4 of compounds for MRSA and all compounds for *Pa* at varying extents (Fig. 1a). Among the combintations, thymol and BL showed the most distinct antimicrobial effect, with a >2 log CFU/mL reductions for all strains tested (Fig. 1a, outlined in red).

We next evaluated any risk of drug-resistance induced by the combined approach with two representative strains: MRSA HS0182 and *Pa* HS0028. The strains remained susceptible to the combined treatment after 20 cycles of sub-lethal selections (Fig. 1b). Conversely, exposure of MRSA HS0182 to penicillin (PEN) or *Pa* HS0028 to ampicillin (AMP) substantially increased the MICs with a 64- or 40-fold MIC elevation for PEN or AMP, respectively, after the 20th passage (Fig. 1c).

### BL and thymol synergistically killed planktonic bacteria, biofilms, and persisters

**Fig. 2:**
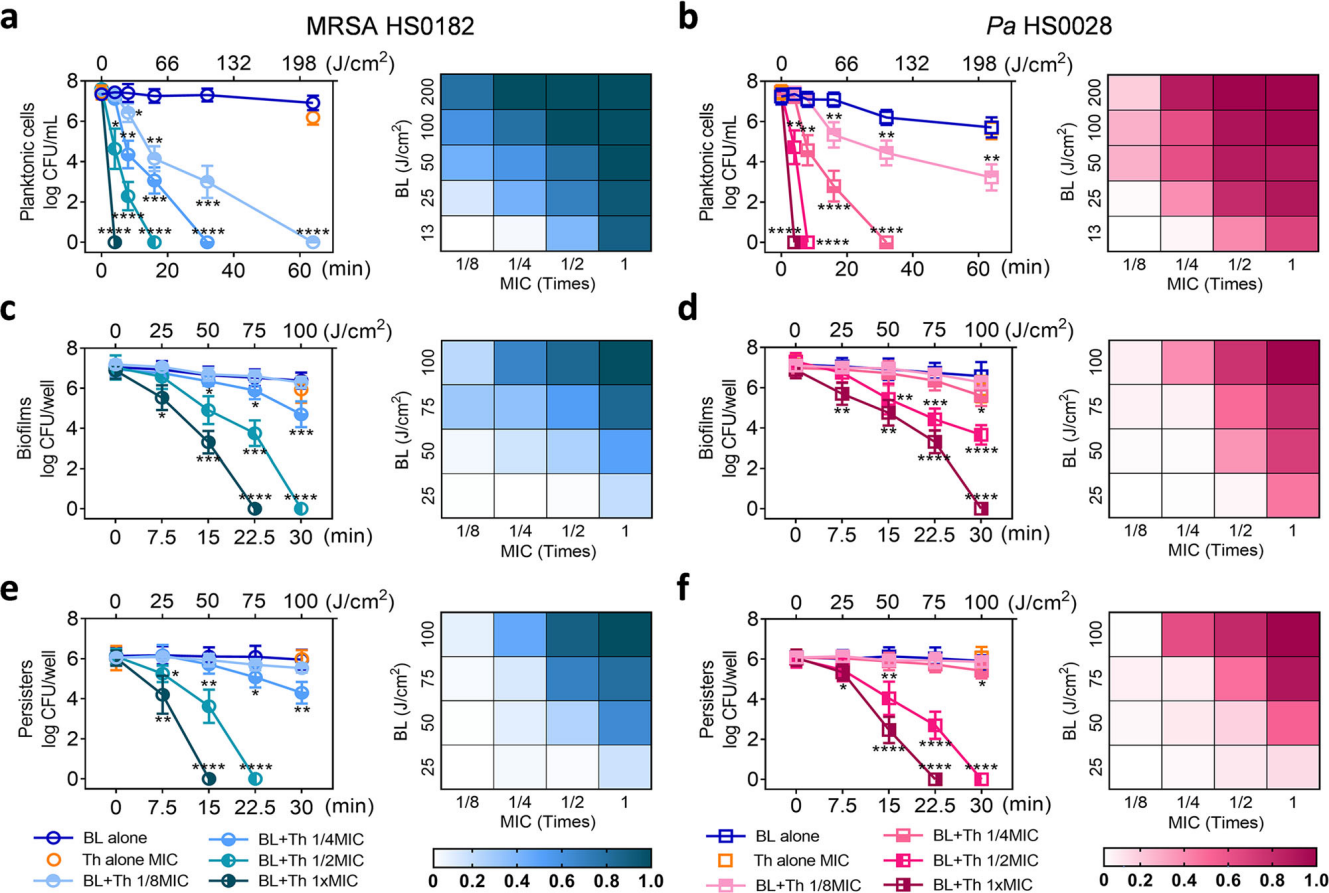
BL and thymol synergistically inactivate planktonic cells, established biofilms, and bacterial persisters. Killing curves (left) and synergy evalution heat map (right) of MRSA HS0182 (**a**, **c**, and **e**) and *Pa* HS0028 (**b**, **d**, and **f**) in planktonic cells (**a** and **b**), established biofilms (**c** and **d**), and bacterial persisters (**e** and **f**) treated with an increasing BL fluence alone, thymol at 1x MIC alone, or BL combined with an increasing concentration of thymol (a fraction of MIC). Checkerboards in the corresponding right panels show *S*-values for different combinations of BL and thymol as assessed by the Bliss Independence model according to the following formula: *S*-value = (logCFU/mL_BL_/logCFU/mL_Control_)(logCFU/mL_Th_/logCFU/mL_Control_) − (logCFU/mL_BL+Th_/logCFU/mL_Control_). LogCFU/mL_BL_, logCFU/mL_Th_, logCFU/mL_BL+Th_, and logCFU/mL_control_ are the number of viable bacteria remaining after treatment with BL alone, thymol alone, combination of BL and thymol, or sham light, respectively. 0 < *S* < 1 indicates a synergistic interaction, whereas *S* < 0 indicates an antagonistic interaction. Results are presented as mean ± SD of four to six replicates from five independent experiments. ****P < 0.0001; ***P < 0.001; **P < 0.01; *P < 0.05; and ns, no significance.

Planktonic MRSA HS0182 (Fig. 2a) and *Pa* HS0028 (Fig. 2b) were synergistically inactivated by BL and thymol, as indicated by the positive *S*-values in the heat maps. *S*-values were calculated according to the Bliss Independence model^12^. The synergies were dose-related. As BL or thymol increased in doses, the *S*-values raised and displayed a dark patch toward the upper right corner where clustered the most effective combinations (Fig. 2a, b; right). Planktonic bacteria at 7.5-log CFU/mL were promptly eradicated by 4-min BL exposure (13 J/cm^2^) in the presence of 1× MIC thymol. In contrast, BL (200 J/cm^2^) or thymol (at sub-MICs) by itself did not severely affect the survival of planktonic cells (Fig. 2a, b; left). The remaining species of the MDR panel were also tested and shown comparably susceptible to the combined BL (30 J/cm^2^) and thymol (1× MIC) treatment, whereas monotherapies were ineffective (Supplementary Fig. S1a). BL and thymol together impaired bacterial envelope integrity, as suggested by propidium iodide (PI) staining (Supplementary Fig. S2). PI^+^ MRSA HS0182 and PI^+^ *Pa* HS0028 surged to 88% and 95%, respectively, after the duo treatment, whereas fewer than 5% PI^+^ cells were seen in monotherapies (*P* < 0.0001).

Mature biofilm formed by MRSA HS0182 (Fig. 2c) or *Pa* HS0028 (Fig. 2d) was also eradicated synergistically by the duo, again with positive *S*-values, though requiring high doses of thymol and/or BL. Thymol (1× MIC) combined with 75-J/cm^2^ or 100-J/cm^2^ BL completely removed viable biofilms of MRSA HS0182 or *Pa* HS0028 (7.0 log CFU/well), whereas BL alone at 100 J/cm^2^ or thymol alone at 1 MIC hardly affected the biofilms (Fig. 2c, d; left). Similar anti-biofilm activities of the combined therapy were also confirmed in other MDR strains listed in Fig. 1a (Supplementary Fig. S1b).

Scanning electron microscopy (SEM) revealed a detrimental effect of the combined therapy on biofilm structures (Supplementary Fig. S3). The biofilm of MRSA HS1082 was patchy and thick in the control group but effaced by the duo treatment. The effacement of MRSA clusters at the upper layers exposed many void matrices and some scattered cells (Supplementary Fig. S3a vs. 3b). For *Pa* HS0028, the orderly polymeric matrices seen in the control group were utterly disrupted by the duo treatment. The remaining bacteria rested sparsely on the bare surface; collapses (arrows) and bacterial wreckages (stars) were around the place (Supplementary Fig. S3c vs. 3d).

Biofilms of MRSA HS0182 or *Pa* HS0028 were next treated with 100× MICs of rifampicin and ciprofloxacin, respectively, for the selections of persisters. It was found that the combined therapy remained effective in killing the dormant persisters. The anti-persister synergies were comparable to the anti-biofilm synergies, as shown by the similar patterns on heat maps (Fig. 2e, f; right). The MRSA HS0182 or *Pa* HS0028 persisters were entirely eradicated by thymol at 1× MICs combined with 50- or 75-J/cm^2^ BL, respectively. Conversely, the persister reductions in monotherapies were <1 log CFU/well (Fig. 2e, f; left). Likewise, similar bactericidal effects of the duo on persisters were confirmed in other MDR strains (Supplementary Fig. S1c).

### Topical application of BL and thymol sterilized wound contamination

**Fig. 3:**
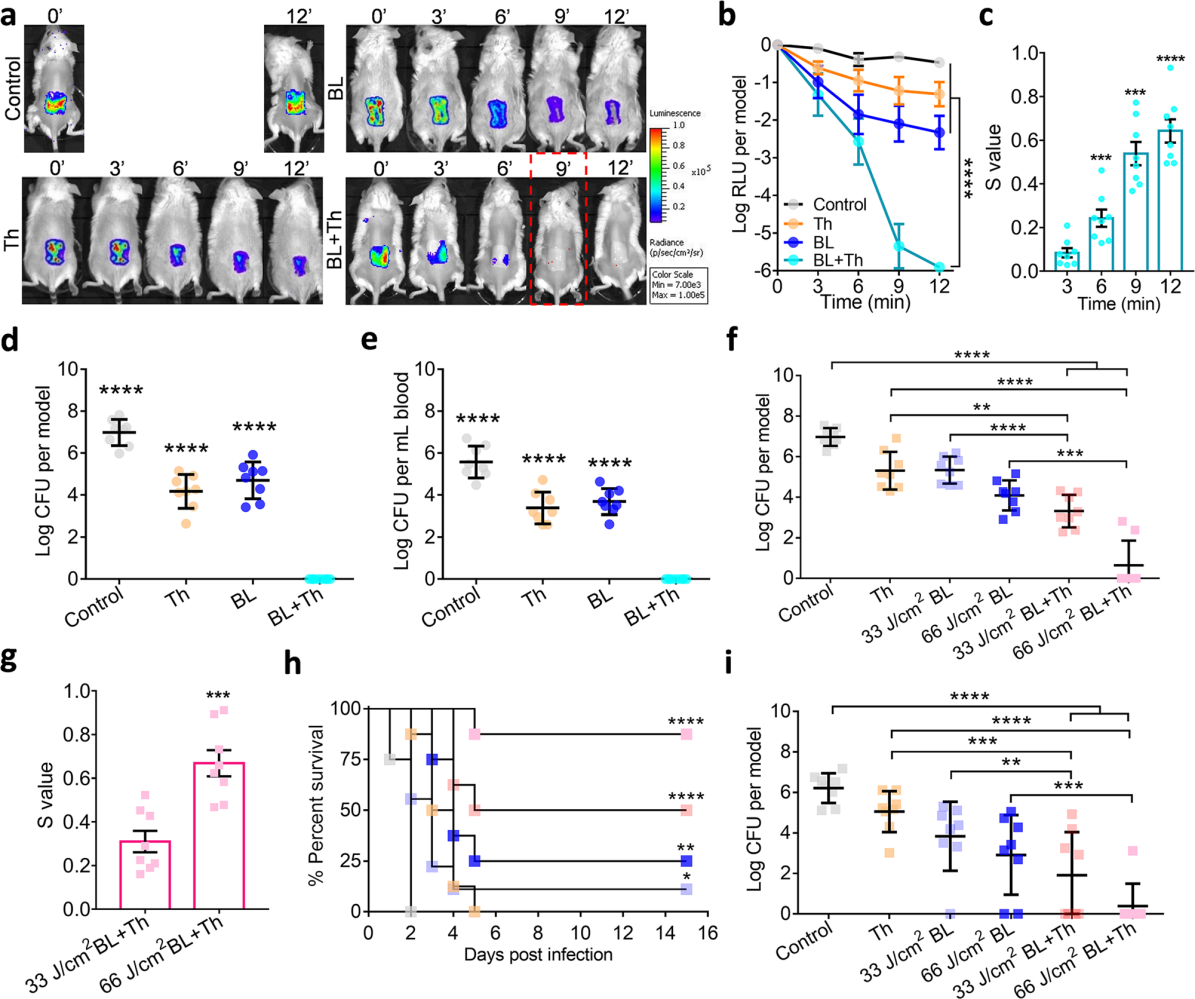
Synergistic disinfections of murine burns by topical application of BL and thymol. Full-thickness 3rd-burn-wounds were infected with USA300 (**a**–**e**) or *Pa* HS0028 (**f**–**i**) at 5 × 10^6^ CFU in 50 μL of PBS for 30 min as acute infection. **a**–**e** The USA300-infected wounds were exposed to sham (control), 50 µL of thymol at 2 mg/mL (Th), indicated times of BL exposure (BL), or both (BL + Th). **a** Bacterial luminescence images of representative wounds were acquired at indicated times after various treatments. **b** Mean luminescence was presented as logarithmic relative luminescence units (log RLU) per model relative to time zero. **c** *S*-values are calculated by the Bliss Independence model as Fig. 2. **d** and **e** Bacterial burdens in the wounds (**d**) and blood (**e**) were assayed 7 days after acute infection shown as log CFU per model. **f**–**i** The *Pa* HS0028-infected burns were treated with sham (control), 50 µL of thymol at 10 mg/mL (Th), BL exposure at 33 J/cm^2^ or 66 J/cm^2^, or both and log CFU per wound were determined on day 1 after the indicated treatments. **g** *S*-values confirm the synergy between BL and thymol against acute *Pa* HS0028 infection. **h** Kaplan–Meier survival curves of *Pa* HS0028-infected mice. **i** Bacterial loads in the burns were quantified either prior death or at the end of the experiments. All results are presented as mean ± SD of eight mice. Zero in **d**, **e**, **f**, and **i** was below the detection limit (40 CFU per wound and 20 CFU per mL blood). ****P < 0.0001; ***P < 0.001; **P < 0.01; and ns, no significance.

Full-thickness 3rd-burns were inflicted and infected by the luminescent USA300 for 30 min, after which the acute contaminated wounds received sham therapy, monotherapies, or combined BL and thymol at 100 µg in 50 µL. Bacterial luminescence signal, as a surrogate for viability, remained stable in the control group. The BL or thymol monotherapy slightly attenuated the luminescence overtime, yet complete elimination of the signal was only achieved by the combined therapy starting at 9-minutes irradiation (30 J/cm^2^) (Fig. 3a; Red dashed box). After quantifying the luminescence signal, a significant reduction was seen in the combination group (Fig. 3b; *P* < 0.0001). The combined therapy’s sterilization effect was proven synergic in all combinations per the Bliss Independence model (Fig. 3c). The combined therapy completely sterilized the wound and successfully prevented bacteremia on day 7. In contrast, the monotherapies reduced bacterial loads at the burns only by 2- to 3-log CFU and failed to halt bacterial invasion (Fig. 3d, e).

Likewise, the burns were acutely infected with *Pa* HS0028 and treated with thymol and/or BL (33 or 66 J/cm^2^) as above (Fig. 3f–i). On day 1, 75% of mice in the combined 66 J/cm^2^ BL and thymol group carried a completely sterile wound (Fig. 3f). Moreover, a higher dose of BL showed more potent synergy with thymol (Fig. 3f, g). The combined groups showed the best survival rate after a course of 15-days infections. The duo with 66 J/cm^2^ or 33 J/cm^2^ BL exposure gave rise to 87.5% and 50% survival, which was significantly higher than 0%, 0%, 12.5%, and 25% survival of mice treated with sham, thymol alone, 33 J/cm^2^ BL alone, and 66 J/cm^2^ BL alone, respectively (Fig. 3h; *P* < 0.0001). On day 15, the percentages of mice with a sterile wound were 12.5%, 25%, 50%, and 87.5% after treatment with 33 J/cm^2^ BL, 66 J/cm^2^ BL, 33 J/cm^2^ BL + Th, and 66 J/cm^2^ BL + Th, respectively (Fig. 3i), which correlated well to the descending CFUs in the corresponding groups on day 1. Therefore, the host may better handle an infection if timely interventions are provided to minimize acute bacterial loads.

### BL together with thymol rescued mice from lethal USA300 biofilm-associated infection

**Fig. 4:**
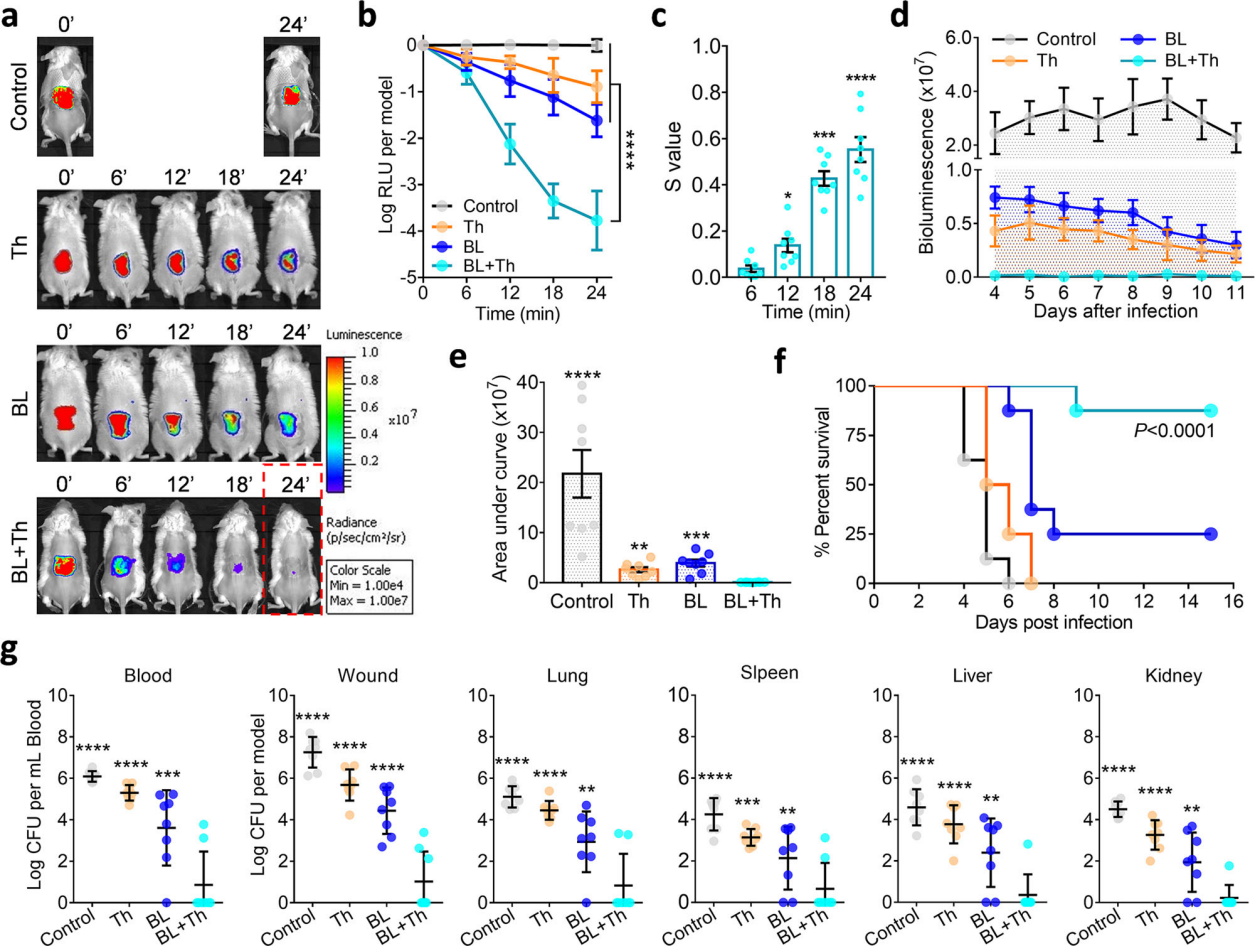
BL together with thymol rescues mice from lethal USA300 biofilm-associated infection. Murine 3rd-burn-wounds were infected with USA300 at 5 × 10^7^ CFU in 50 µL of PBS for 72 h to form mature biofilms. The infected wounds were treated with sham (control), 50 µL of thymol at 10 mg/mL (Th), indicated times of BL (BL), or both (BL + Th). **a** Bacterial luminescence images of representative wounds were acquired at indicated times. **b** Mean luminescence was acquired over time and presented as log RLU per model. **c**, *S*-values are calculated by the Bliss Independence model as Fig. 2. **d** and **e** Mean luminescence was acquired from days 4 to 11 after an indicated treatment and the mean areas under the luminescence curves were summarized in **e**. **f** Kaplan–Meier survival curves of USA300 biofilm-associated mice in response to an indicated treatment. **g** Bacterial loads in the blood, wounds, lungs, spleens, livers, and kidneys were quantified just prior death or on day 15 after bacterial inoculation. All results are presented as mean ± SD of eight biological replicates. Zero in **g** was below the detection limit (40 CFU per model for murine wounds or organs and 20 CFU per mL blood). ****P < 0.0001; ***P < 0.001; **P < 0.01; and ns, no significance.

The burns were infected with USA300 for 72 h to allow biofilm establishment. The biofilm-associated wounds were then treated with sham therapy, monotherapies, or combined BL and thymol as above. Monotherapies slightly suppressed the luminescence overtime, yet virtual elimination of the bioluminescent signal was only attained by the combined therapy at 24-minutes irradiation (80 J/cm^2^) (Fig. 4a; Red dashed box). The combined therapy’s anti-biofilm activity at 24 min was 750 or 140-folds more potent than thymol alone or BL alone, respectively (Fig. 4b; *P* < 0.0001). The synergy between BL and thymol against USA300 biofilm-associated infection was confirmed by the Bliss Independence model (Fig. 4c). We traced the bacterial luminescence on wounds for 7 days after treatments (days 4 to 11) (Fig. 4d). No rebound of bacterial growth was found at the wounds in the combined group (Fig. 4e). Moreover, the combined therapy saved 87.5% of mice by day 15, which was significantly higher than 0%, 0%, or 25% survival of mice treated with sham, thymol alone, or BL alone, respectively (Fig. 4f; *P* < 0.0001). Most importantly, the combined therapy prevented systematic dissemination of USA300, whereas the monotherapies failed. In support of this, notably fewer bacteria were recovered from the blood and vital organs (i.e., lung, spleen, liver and kidney) in the combined group (Fig. 4g).

### The combined therapy exhibited no adverse effects on fibroblasts and mouse skins

**Fig. 5:**
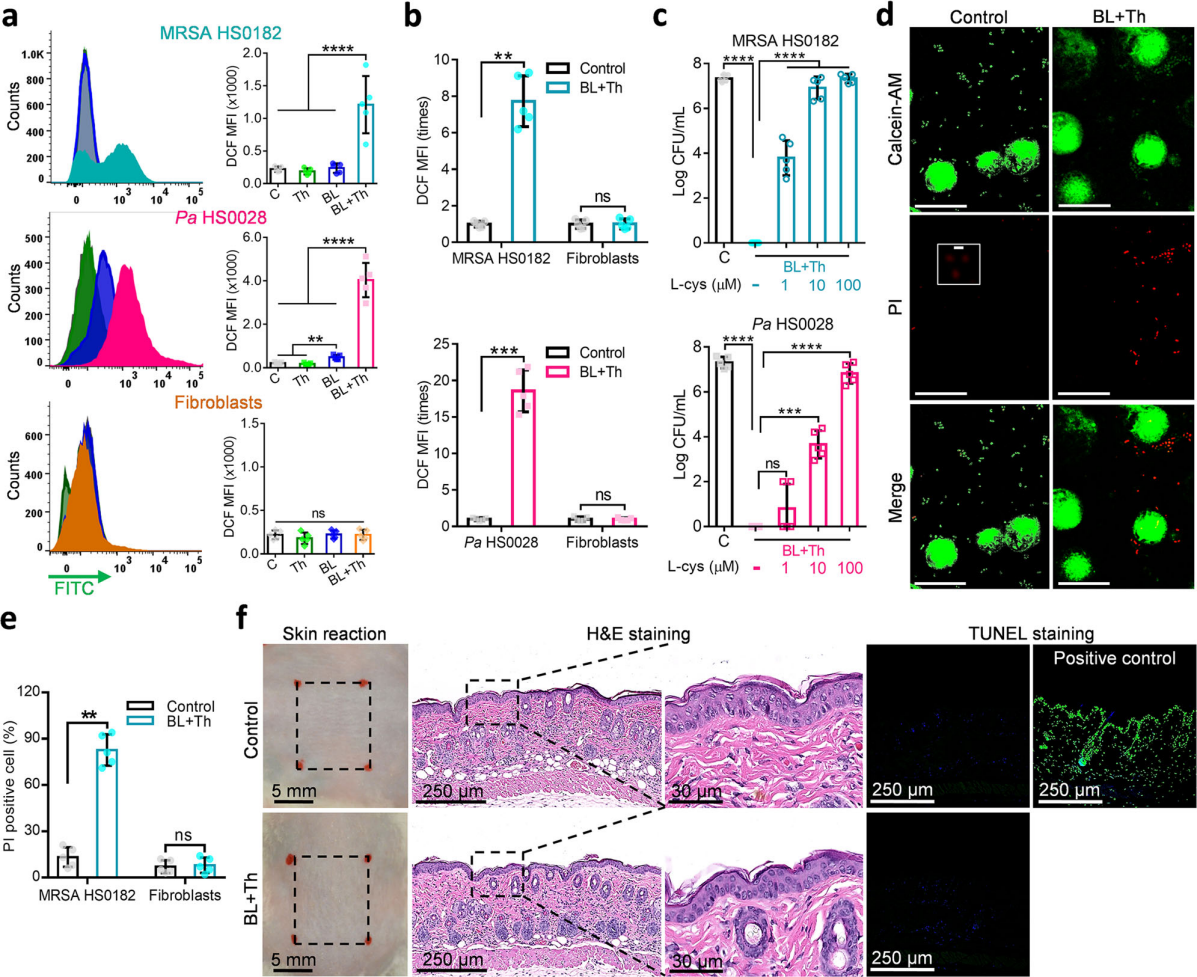
No adverse effects of BL combined with thymol treatment on fibroblast and murine skin. **a** Flow cytometric analyses of intracellular ROS in MRSA HS0182 (upper), *Pa* HS0028 (medium), and fibroblasts (bottom) treated with sham light (C), thymol at 1/2 MIC (Th), an indicated BL fluence (BL), or both (BL + Th) by DCFH-DA fluorescent staining. MRSA HS0182 was exposed to thymol at 0.15 mg/mL, BL at 50 J/cm^2^, or both, and *Pa* HS0028 to thymol at 0.3 mg/mL, BL at 25 J/cm^2^, or both. Alternatively, fibroblasts alone (**a**, bottom) or the cells co-cultured with MRSA HS0182 (**b**, upper) or *Pa* HS0028 (**c**, bottom) were treated with thymol at 0.5 mg/mL, BL at 50 J/cm^2^, or both. DCF mean florescence intensity (MFI) on gate of bacteria or fibroblasts was presented in **a** and fold changes of DCF MFI relative to sham-treated controls were shown in **b**. **c** Dose-dependent effects of antioxidant L-cys on the bactericidal activity of the combined therapy: 50 J/cm^2^ BL and 0.15 mg/mL thymol for MRSA HS0182 and 25 J/cm^2^ BL and 0.3 mg/mL thymol for *Pa* HS0028. **d** Representative fluorescence images of co-culture of MRSA HS0182 and fibroblasts were shown, in which the dead and viable cells were visualized by PI (red) and calcein-AM staining (green), respectively. Scale bars, 20 µm. An area in the middle panel (control PI) was enlarged to show a few PI-stained bacteria (Scale bars, 2 µm). **e** PI^+^ MRSA HS0182 and PI^+^ fibroblasts in co-cultures were also counted manually and presented as percentages relative to a total of cells. **f** No adverse effect of topical application of BL and thymol in murine skin. The dorsal skin was topically treated with sham (control) or 100 J/cm^2^ BL and 50 µL of thymol at 20 mg/mL (BL + Th) once a day for 5 consecutive days. On day 6, the skins were processed by H&E histological examination and TUNEL assay. DNase I-treated skins were TUNEL stained in parallel as positive-staining controls. All results are presented as mean ± SD of at least five biological replicates. Images in **d** and **f** are representative of five independent experiments. ****P < 0.0001; ***P < 0.001; **P < 0.01; and ns, no significance.

The combined therapy induced ROS production in MRSA HS0182 (upper) and *Pa* HS0028 (middle), but not in human fibroblasts (bottom), as shown by DCF staining (Fig. 5a). In co-culture experiments, where bacteria and fibroblasts received treatments at the same petri dish, the duo induced a 8-fold or 19-fold increase of DCF fluorescence in MRSA HS0182 or *Pa* HS0028, respectively. No notable changes in DCF fluorescence were observed in co-cultured fibroblasts, suggesting that ROS were produced and well trapped within bacterial cells (Fig. 5b). L-cys, a well-recognized ROS scavenger, dose-dependently abrogated the combined therapy’s bactericidal activity in both MRSA HS0182 and *Pa* HS0028, arguing strongly for ROS-dependent bactericide (Fig. 5c). Bacteria-specific killing in the co-culture by BL and thymol was visualized via Calcein-AM/PI staining under a confocal microscope (Fig. 5d). PI^+^ MRSA significantly increased to 81% after the combined therapy, whereas PI^+^ fibroblasts remained at a low level (<10%) before and after treatment (Fig. 5e; *P* < 0.01). After murine skin received five consecutive doses of concentrated thymol (20 mg/mL) and intense BL irradiation (100 J/cm^2^), its structure and integrity were well-preserved (Fig. 5f), confirming that the duo treatment did not adversely affect host cells. The lining of epidermis and dermis after treatment were clear and complete, resembling that of the control group. TUNEL-positive cells were non-detectible, indicating absence of DNA breaks or apoptotic cells in the treated skins (Fig. 5f).

### Photooxiation of thymol into photosensitizers amplifies bactericidal ROS generation

**Fig. 6:**
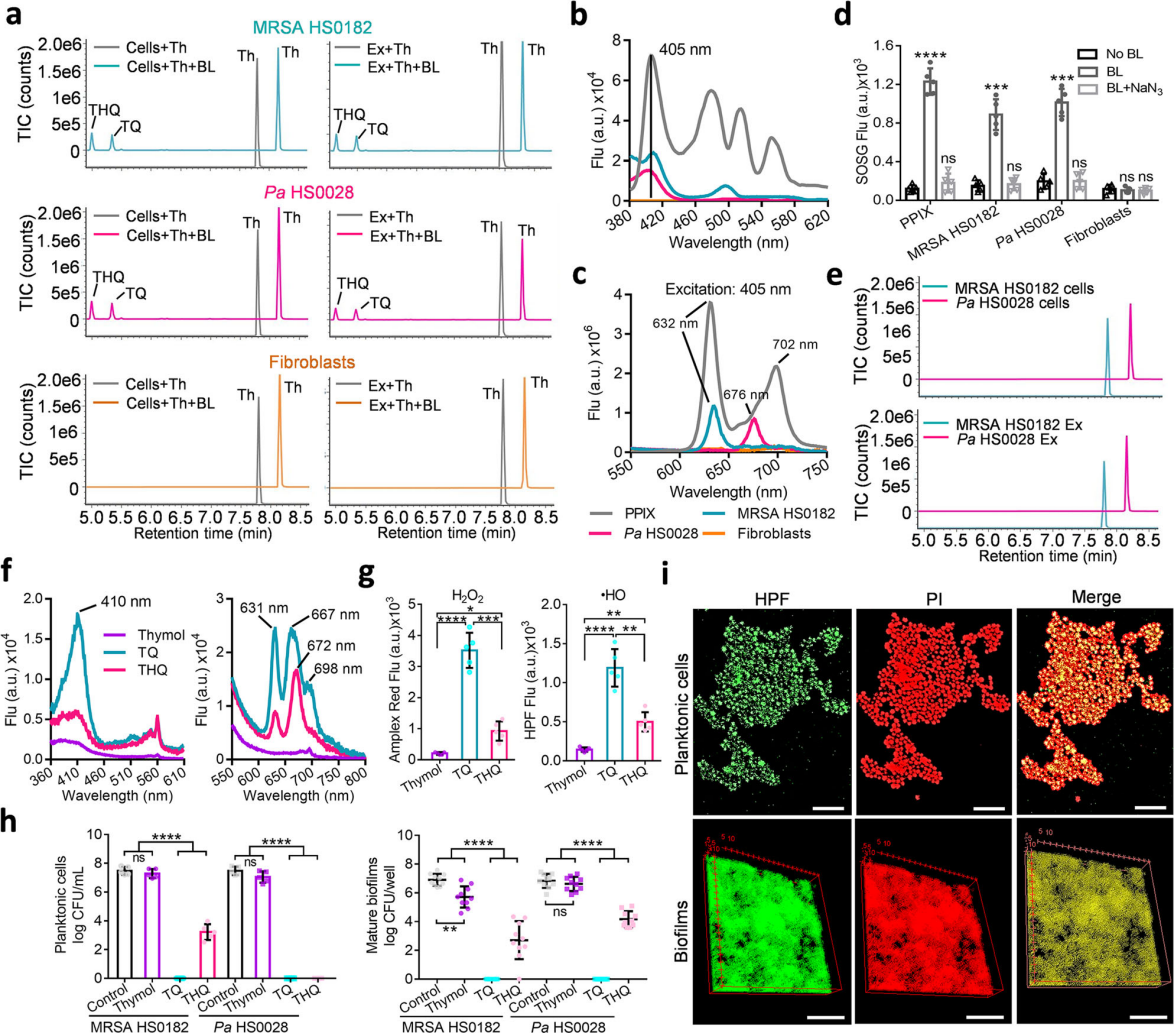
Photo-oxidation of thymol to generate photosensitizers TQ and THQ exclusively in bacteria. **a** UPLC-VION-IMS-QTOF-MS/MS analyses of thymol oxidation in cells (left) or extracts (right) of MRSA HS0182 (upper), *Pa* HS0028 (medium), or fibroblasts (bottom) treated with 0.5 mg/mL thymol in the absence or presence of 50 J/cm^2^ BL exposure. **b** and **c** Exciation (**b**) and emission (**c**) spectra of an indicated cellular extract in comparison with those of PPIX at 10 µM. **d** ^1^O_2_ is notably generated by 50 J/cm^2^ BL in MRSA HS0182, *Pa* HS0028, and PPIX solution but not in fibroblasts. ^1^O_2_ generation was blunted by NaN_3_ (BL + NaN_3_), a ^1^O_2_-specific quencher. **e** UPLC-VION-IMS-QTOF-MS/MS analyses of photo-oxidized thymol in the presence of NaN_3_ at 10 µM. BL illumination at 50 J/cm^2^, thymol at 0.5 mg/mL. **f** Exciation (left) and emission (right) spectra of thymol, TQ, and THQ each at 0.5 mg/mL. **g** H_2_O_2_ and •HO were generated by thymol, TQ, and THQ each at 0.2 mg/mL in combination with 50 J/cm^2^ BL. **h** Bactericidal activities of thymol, TQ, and THQ against planktonic cells (left) and established biofilms (right) of MRSA HS0182 and *Pa* HS0028 in the presence of 20 J/cm^2^ BL. The concentrations of three compounds were same at 0.05 mg/mL for planktonic cells and 0.1 mg/mL for established biofilms. **i** Representative fluorescence images of planktonic cells (upper) and established biofilms (bottom) of MRSA HS0182 after a lethal dose of the duo treatment. Bacterial viability and intracellular •HO were evaluated by PI and HPF staining, respectively. Scale bars: 2 µm in upper panel and 50 µm in bottom panel. All images in **a**, **b**, **c**, **e**, **f**, and **i** are representative of five independent experiments. Results in **d**, **g**, and **h** are presented as mean ± SD of at least five independent experiments. ****P < 0.0001; ***P < 0.001; **P < 0.01; *P < 0.05; and ns, no significance.

The selectivity of the combined therapy and the bacteria-specific production of ROS directed us to investigate the fate of thymol in the presence of BL. As shown by ultra-performance liquid chromatography-VION-ion mobility spectrometry-quadrupole time-of-flight-tandem mass spectrometry (UPLC-VION-IMS-QTOF-MS/MS), thymol was oxidized to TQ and THQ in viable MRSA HS0182 (upper) and *Pa* HS0028 (middle) or their extracts after BL exposure, whereas fibroblasts (bottom) or it extracts failed to convert thymol in the presence of BL (50 J/cm^2^) (Fig. 6a). Chromatograms and mass spectra of thymol, TQ, and THQ standards were run in parallel to confirm the chemical compounds (Supplementary Fig. S4). Next, we compared the excitation and emission spectra of MRSA HS0182, *Pa* HS0028, and fibroblast extracts with those of protoporphyrin IX (PPIX), an endogenous blue-laser sensitizer. All bacterial extracts had excitation at 405 nm like PPIX, while such excitation peak was absent in fibroblast extract (Fig. 6b). In response to 405-nm BL, MRSA HS0182 extract emitted predominantly at 632 nm, and *Pa* HS0028 extract at 676 nm, similar to the PPIX emission peaks at 632 nm, 668 nm, and 702 nm, whereas no emission was seen in fibroblast extract (Fig. 6c). Production singlet oxygen (^1^O_2_) is a hallmark of PPIX photo-excitation. After BL exposure, ^1^O_2_ was detectable in MRSA HS0182, *Pa* HS0028, and PPIX solution, but not in fibroblasts, in agreement with negligible BL sensitizers in mammalian cells (Fig. 6d). Of note, the generation of ^1^O_2_ in bacterial cells or PPIX solution were canceled by the ^1^O_2_ quencher NaN_3_ (Fig. 6d). In line with this, the oxidative transformation of thymol into TQ and THQ in bactreia cells (upper) and their extracts (bottom) was also abrogated by NaN_3_ (Fig. 6e). These results confirmed that proporphyrin-like compounds in bacteria and the subsequent ^1^O_2_ generation were vital for the transformation of thymol into a photosensitizer.

We further discovered that the oxidized products, TQ and THQ, were by themselves BL-sensitizing agents. Both TQ and THQ, particularly TQ, exhibited an excitation peak at around 410 nm, while thymol showed none of such excitation (Fig. 6f; left). TQ and THQ also exhibited a similar emission peak at about 630 nm and 670 nm in response to 405-nm BL, measured by a fluorescence spectrometer (Fig. 6f; right). Accordingly, TQ and to a lesser extent THQ, rather than thymol, generated substantial amounts of H_2_O_2_ and •HO upon BL irradiation (Fig. 6g). We suspected that TQ and THQ, most likely TQ, contributed notably to the ROS source during the combined therapy. In support, at low doses of BL (20 J/cm^2^) and compounds (0.05-0.1 mg/mL), TQ and THQ were significantly more potent than thymol in killing planktonic or biofilm bacteria (Fig. 6h; *P* < 0.0001). Furthermore, •HO producing planktonic/biofilm bacteria (green; HPF^+^) overlapped perfectly with dying bacteria (red; PI^+^), which validated that the combined therapy inactivated bacteria through the action of ROS, particularly by the detrimental •HO (Fig. 6i).

## Discussion

This study unravels a previously unappreciated “pro-photosensitizer” function of thymol that is activated exclusively in bacteria upon BL illumination. The unexpected finding paves an innovative strategy to seek bacteria-specific pro-photosensitizers with which more effective and specific non-antibiotic modalities can be developed to combat the crisis of MDR superbugs. MDR microbes are commonly colonized on the wound surface, especially on chronic skin wounds, which contaminate the healthcare environment frequently and readily spread the bacteria to vulnerable patients in hospitals because these wounds are openly exposed to the atmosphere. When the body surfaces are disinfected quickly, safely, and repeatedly by a modality, it not only benefits patients greatly but also effectively eliminates the sources of nosocomial infections, making the healthcare environment safe to vulnerable patients. Moreover, the modality is not limited to skin wounds and can be potentially extended to disinfect other tissues such as urinay tract, throat, and mouth, surgical sites, tooths, and so on.

Antimicrobial BL and essential oils both possess a multi-target mode of action and broad antimicrobial spectra. BL at 400 to 495 nm has been shown to be bactericidal to an array of pathogens through the generation of ROS. ROS indiscriminately damages cellular components (e.g., lipids, proteins, plasma membrane, and nucleic acids). Likewise, essential oils act on multiple bacterial targets, particularly the bacterial envelopes^8,13,14^. Thus, repeated exposures to BL and essential oils are unlikely to render bacterial tolerance^8,9,15–17^. Essential oils are comprised of volatile constituents produced by aromatic plant/herbs. Among 3000 essential oils, about 300 are generally recognized as safe (GRAS) to humans by the United States Food and Drug Administration (U.S. FDA) and have broad applications in food preservation, additives, favors, perfume, cosmetic industries, antiseptic oral solutions, toothpastes, cleaner, and air fresheners for centuries^18,19^. In attempt to develop non-antibiotic alternatives, we have screen a few dozens of the essential oils for their ability to kill MDR bacteria^8^. We found potent antimicrobial activity of the volatile oil prepared from *Thymus vulgaris*, which was also confirmed by other groups^20,21^. The predomiant component of the volatile oil is thymol (47.59%) and exhibited strong synergy with BL, whereas compounds isolated from other essential oils appeared to be weak or no such synergy, including eugenol, cinnamaldehyde, cuminaldehyde, citral-a, terpinen-4-ol, and menthol, although these essential oil compounds possess similar antimicrobial activity as thymol (Fig. 1a).

**Fig. 7:**
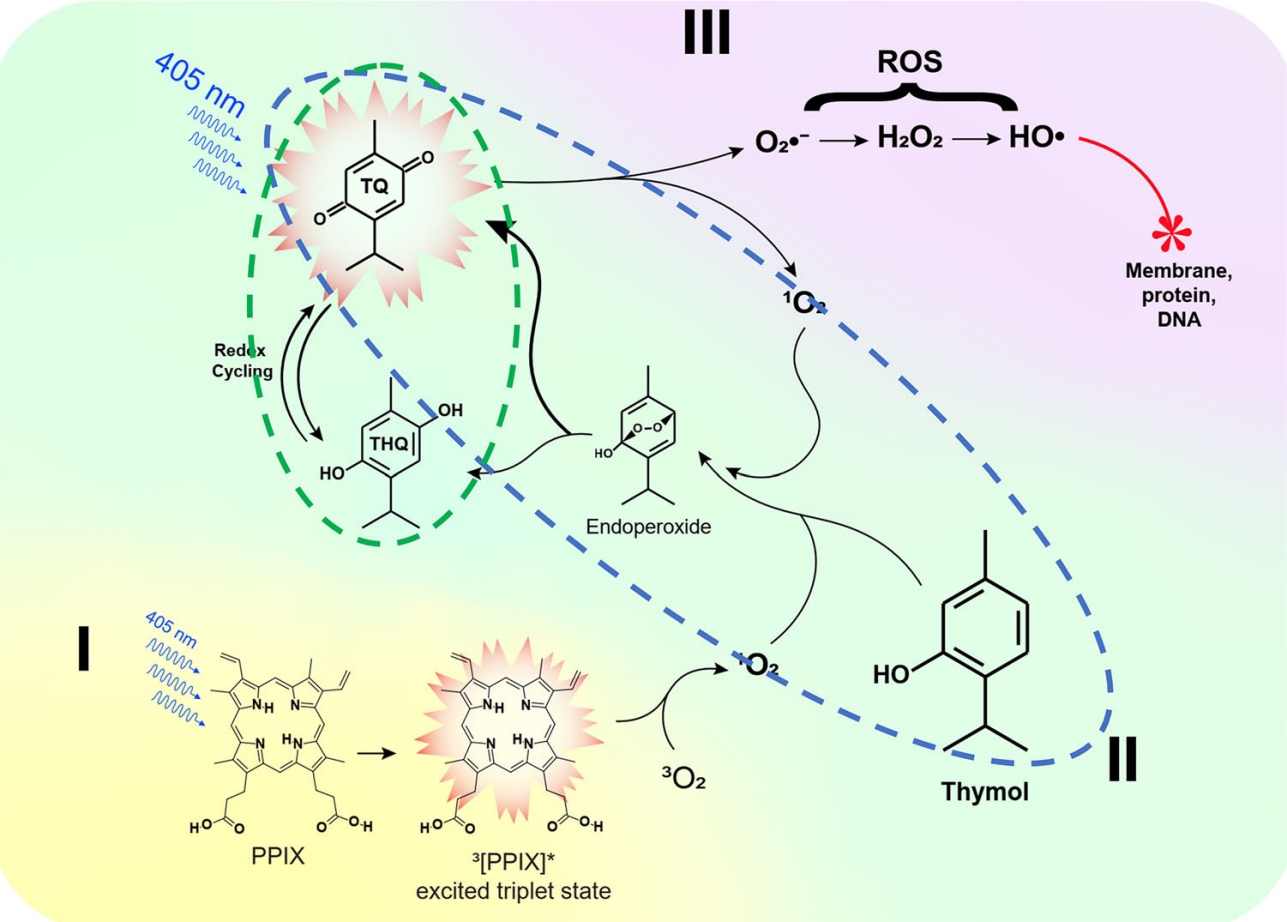
A schematic of phototoxic cascade reactions in the presence of BL and thymol. Endogenous proporphyrins-like compounds absorb BL to attain an excited triplet-state from an electronic ground state and react with a ground state molecular oxygen (^3^O_2_) to generate singlet oxygen (^1^O_2_) (**I**). The ^1^O_2_ oxidizes thymol to form TQ and/or THQ via an endoperoxide intermediate (**II**). The resultant TQ and THQ, particually, TQ act as a photosensitizer and generates more O_2_•^−^ and ^1^O_2_ that can in turn oxidize thymol, continuously replenishing the TQ pool, which forms the first autoxidation cycle (blue-dashed outline). The second autoxidation cycle comprises THQ oxidation into TQ that can be then photo-hydrolyzed into THQ (green dashed outline). O_2_•^−^ undergoes dismutation and forms H_2_O_2_ that is converted into the most detrimental •HO via a Fenton reaction or photolysis (**III**). The deleterious •HO initiates a chain reaction that oxidatively damages the bacteria.

Intriguingly, thymol and BL selectively targeted bacteria while sparing the surrounding host tissue, as confirmed in vivo histologically, as well as by DNA damage assays and cell viability and ROS production in co-cultures of bacteria and fibroblasts (Fig. 5). The bacteria-specific photochemical reaction is likely triggered by BL excitation of endogenous proporphyrins-like compounds in bacterial cells. The excited triplet-state proporphyrin-like compounds collide with molecular oxygen (^3^O_2_) generating highly reactive ^1^O_2_^22–24^ (Fig. 7). This photochemical reaction occurred in MRSA or *Pa* cells or their extracts but not in mammalian cells or their extracts (Fig. 6b–d), in agreement with relatively abundant proporphyrin-like compounds generated in bacteria over mammalian cells. Bacteria spontaneously accumulate tetrapyrrole macrocycles, such as protoporphyrin, uroporphyrinogen III, coproporphyrinogen III, coproporphyrin III, etc. which are BL-sensitive photosensitizers in the basis of their absorbance and excitation spectra and ability to respond to BL similarly as PPIX (Fig. 6). These precursors were generated during synthesis of various metallated tetrapyrroles in bacteria essential for their survival in various environments^25^. The derivatives vary with bacterial strains or isolates and living conditions and are quite complex, identification of which is beyond the scope of the current investigation^23,25–27^.

As depicted in Fig. 7, ^1^O_2_ generated from photo-excitation of endogenous proporphyrins-like compounds presumably oxidized thymol to TQ and THQ in bacteria as shown in Fig. 6a. Photo-oxidation of the phenolic hydroxyl group to para-benzoquinone by ^1^O_2_ was previously confirmed^28^. Photo-conversion of thymol to TQ in the presence of porphyrins was also described elsewhere^29^. The resulting TQ and to a lesser extent, THQ were BL-sensitizers; they produced substantial amounts of H_2_O_2_ and •HO once irradiated (Fig. 6g). Apparently, in this combined treatment, thymol functions as a “pro-photosensitizer” analogous to a prodrug, which is harmless at sub-MICs for the bacteria until BL converts it to TQ and THQ. Like other quinones, TQ can be reversibly transformed into semiquinone and THQ via redox cycling^30^, forming an autoxidative cycle. TQ is subsequently excited by BL and generates either superoxide anion (O_2_•^−^) or ^1^O_2_ via Type I or Type II photo-oxidation. ^1^O_2_ continuously reacts with thymol and maintains the pool of TQ and THQ, giving rise to another autoxidation cycle. The two autoxidation cycles interacted with each other exponentially amplying ROS production over time as long as BL is presented. O_2_•^−^ undergoes dismutation and forms H_2_O_2_ and O_2_. H_2_O_2_ may be rapidly converted into the most detrimental •HO either in a Fenton reaction or by photolysis^31–33^. The deleterious •HO initiates a torrent of cytotoxic events that oxidatively damage the fatty acids, lipids, amino acids, and nucleobases (Fig. 7). As ^1^O_2_ and •HO are highly reactive and short-lived, they cause oxidative damages to proximal biomolecules before they can escape the bacterial cells. Of note, none of the above events occur in mammalian cells due to insufficient metal-free proporphyrins-like substances to generate sufficient ^1^O_2_ (Fig. 6d). TQ has been widely tested in various medinces as antioxidant^34^ and it is harmless in the absence of BL, but it is toxic in the presence of BL as it is a photosensitizer. Various photosensitizers are used for antimicrobial photodynamic therapy (aPDT), in which a photosensitizer enters not only bacteria but also mammalian cells and generates ROS similarly in these two types of cells so that killing bacteria over mammalian cells depends on relative susceptibility to ROS between bacteria and mammalian cells and finding a safe and effective window of aPDT could be challenging. In sharp contrast, thymol behaves as a pro-photosensitizer and is converted into an active photosensitizer by BL exclusively in bacteria. Conceivably, pro-photosensitizers like prodrugs ensure specificity and safety and represent notable advantages over traditional aPDT.

Limitation of the modality lies in poor tissue penetration of BL and inability to decomtaminate deep tissue. Moreover, some bacteria like *Escherichia coli* produce latively low amounts of BL-sensitive tetrapyrrole macrocycles and may not be susceptible to the combinatory treatment^35,36^. Thus, further studies are needed to enhance bacteria-specific ROS generation, which can then convert harmless pro-photosensitizers like thymol into photosensitizers exclusively in most of bacterial pathogens.

In summary, the combined BL and thymol induced oxidative burst exclusively within bacteria. The confined ROS rapidly and selectively inactivated all forms of bacteria, including planktonic cells, biofilms, and persisters, regardless of their antibiotic-resistance profiles. In accordance with this, the combined topical therapy synergistically sterilized acutely infected or biofilm-associated wounds and effectively prevented subsequent bacterial invasion or dissemination in mice while incurring no adverse effects on host cells. This highly selective modality is less likely to develop resistance and can serve as an alternative to antibiotics to frequently and repeatedly disinfect skin wounds or body surfaces and prevent sepsis, particully useful to treat chronic skin wounds. A combination of BL and thymol thus holds promise as a safe, attractive, and non-antibiotic therapeutic in fighting MDR bacteria.

## Methods

### Light source, compounds, microorganisms, and cell lines

A light-emitting diode (LED, Thorlabs) with peak emission at 405 nm and a full width at half maximum of 12.5 nm was used. The irradiation was fixed to obtain 55 mW/cm^2^ by altering the distance of the light source aperture and the target surface with the use of a PM100D power/energy meter (Thorlabs). A small soft white LED bulb (3 W, A15) from General Electric was used as sham light at a similar intensity. Phytochemical compounds (>98% purity) listed in Fig. 1 were purchased from Sigma-Aldrich. The stock of every compound was prepared at 50 mg/mL in N, N-Dimethylformamide. The clinical strains of MRSA (RJ0021, RJ0056, HS0006, and HS0182) and *Pa* (RJ0002, RJ0006, HS0001, and HS0028) were isolated from patients with burn infections from Huashan Hospital and Ruijin Hospital. A luminescent strain of USA300 was used for real-time monitoring skin infection via bioluminescence imaging^9^. The bacteria were routinely cultured overnight at 37 °C on brain heart infusion (BHI) agar plate supplemented with 5% sheep blood, followed by additional 3 or 20 h culture at 37 °C at 180 rpm in BHI broth to obtain a mid-logarithmic or stationary growth-phase, respectively. The fibroblasts were purchased from ATCC (ATCC PCS-201-012) and cultured for 2–3 days at 37 °C with 5% CO_2_ in Dulbecco’s modified Eagle’s medium (DMEM) supplemented with 10% fetal bovine serum (FBS), 100 units/mL penicillin, and 100 µg/mL streptomycin.

### Bactericidal activity against planktonic cells, biofilms, and persisters

A stationary growth-phase culture at 5 × 10^7^ CFU/mL in PBS was added to 35-mm Petri dishes at 2970 µL, along with a final concentration of thymol at 1/8, 1/4, 1/2, and 1× MIC at 30 µL, followed immediately by BL exposure while stirring at 60 rpm. Aliquots of 50 µL of the suspensions were withdrawn at 0, 4, 8, 16, 32, and 64 min, corresponding to 0, 13, 25, 50, 100, and 200 J/cm^2^ BL. The bactericidal effects were similarly assessed with BL alone, thymol alone or thymoquinone (TQ) or thymohydroquinone (THQ) either alone or in combination with 20 J/cm^2^ BL. CFU were determined by 10-fold serial dilutions on BHI agar plates and enumerated after 24-h culture^37^.

A mid-logarithmic growth-phase culture was prepared at 1 × 10^6^ CFU/mL in trypticase soy broth (TSB) containing 0.1% glucose, 1 mM MgSO_4_, 0.15 M ammonium sulfate, and 34 mM citrate^38^. The inoculums were seeded into 96-well plates at 100 μL/well and incubated for 72 h to form biofilms. The formed biofilms were washed twice with PBS and then immersed into 200 µL of thymol solution at a final concentration of 1/4, 1/2, or 1× MIC, followed by BL exposure at 25, 50, 75, or 100 J/cm^2^. Bactericidal effects of BL alone, thymol alone, or TQ and THQ in the presence or absence of 20 J/cm^2^ BL were investigated similarly. The biofilm-encased bacteria were dislodged in 200 µL of PBS by 5-minute sonication for CFU assays as described above. Sonication itself would not affect bacterial viability.

To prepare bacterial persisters, a mid-logarithmic growth-phase culture was prepared in the above-modified TSB medium at 1 × 10^6^ CFU/mL and added to 96-well plates at 200 µL/well. After 72 h of incubation and two washes with PBS, the biofilms were treated with 200 µL of BHI broth containing 100× MICs of rifampicin (1500 µg/mL) for MRSA and ciprofloxacin (800 µg/mL) for *Pa*^39^. The antibiotic was washed out after 24 h of incubation and adherent bacteria were dislodged into 200 µL of PBS. The bacteria surviving the antibiotic treatment were collected as persisters and treated with thymol alone at a final concentration of 1/4, 1/2, or 1× MIC, BL alone at 25, 50, 75, or 100 J/cm^2^, or both, followed by CFU quantification as above.

### Treatment of murine burns infected with USA300 or Pa HS0028

All animal protocols were approved by the Shanghai Jiao Tong University Animal Study Committee. BALB/C mice at 8 weeks of age were anesthetized with an intraperitoneal injection of a ketamine-xylazine cocktail and shaved on the lower dorsal skin. A full-thickness 3rd-degree burn was generated by a brass block (1 cm^2^) heated to thermal equilibration with boiling water prior to the application of its extremity onto the skin for 7 s^26^. Sterile saline was intraperitoneally administered at 0.5 mL/mouse to sustain fluid balance during recovery. Ten minutes after the injury, a stationary growth-phase culture of USA300 or *Pa* HS0028 at 5 × 10^6^ in 50 µL of PBS was incubated onto the burns for 30 min as acute burn infection. Subsequently, the USA300- or *Pa* HS0028- infected burns were smeared evenly with 50 µL of thymol at 2 mg/mL or 10 mg/mL, respectively, and exposed immediately to BL for indicated times. The bactericidal effects were evaluated similarly with BL alone, thymol alone, or vehicle PBS combined with sham light for comparisons. In addition, the burns were infected with USA300 at 5 × 10^7^ in 50 µL of PBS for 72 h to form biofilms in the burns as a lethal biofilm-associated burn infection^26^. Then, the infected burns were treated with sham light, 50 µL of thymol at 10 mg/mL, an indicated time of BL, or both as above.

The bioluminescence imaging of bacteria in wound was performed by using a Lumina II In Vivo Imaging System (IVIS, PerkinElmer). During imaging, mice were anesthetized in the chamber supplemented with 2.0% isoflurane inhalant mixed with oxygen via an IVIS manifold placed within the imaging chamber. Bioluminescence was quantified with the Living Image software (Xenogen). For measurement of bacterial burden, the mice were euthanized at the end of the experiment and perfused with PBS via the heart. The burned skins, lungs, spleens, livers, and kidneys of mice were excised and homogenized in 2 mL of PBS. The resultant homogenate of each tissue was spotted onto BHI agar plate containing Skirrow’s supplements after serial dilutions and processed for bacterial enumeration. At least eight mice were used for each test group.

### Measurements of intracellular ROS in bacteria and fibroblasts

A stationary growth-phase culture of MRSA HS0182 or *Pa* HS0028 at 5 × 10^7^ CFU/mL was treated with a lethal dose of the combined treatment. MRSA HS0182 was treated with thymol at 0.15 mg/mL, BL at 50 J/cm^2^, or both; *Pa* HS0028 was exposed to thymol at 0.3 mg/mL, BL at 25 J/cm^2^, or both. To determine intracellular ROS in fibroblasts or the cells co-cultured with the bacteria, fibroblasts were cultured for 2–3 days at 37 °C with 5% CO_2_ in DMEM supplemented with 10% FBS and antibiotics. The fibroblasts were mixed with bacteria at a cell to bacteria ratio 1:10 and the co-cultures and fibroblasts alone were treated with thymol at 0.5 mg/mL, BL at 50 J/cm^2^, or both. After various treatments, the bacteria and/or fibroblasts were stained with 10 µM DCFH-DA solution per the manufacturer’s instruction and analyzed by flow cytometry (BD Biosciences). In some experiments, l-cysteine (L-cys) was added to the bacterial cultures at indicated concentrations for 30 min prior to treatment. All flow cytometric data were analyzed by FlowJo software.

### Cell viability

The co-culture of fibroblasts and bacteria were treated with sham light or a lethal dose of the combined modality (50 J/cm^2^ BL and 0.5 mg/mL thymol). Then, the co-cultures were stained with 10 µM of calcein-AM solution to identify viable cells and 10 µM of PI solution to mark dead cells per the manufacturer’s instruction. The cells were imaged with FluoView FV1000-MPE confocal microscopy (Olympus) via the green/red fluorescence channel.

### Determination of excitation and emission spectra of extracts prepared from fibroblasts and bacteria

Fibroblasts were cultured as above, after which the confluent cells were collected, washed, and resuspended in PBS. MRSA HS0182 and *Pa* HS0028 were incubated for 20 h at 37 °C in BHI broth, then washed twice with PBS, and resuspended in PBS. Total protein levels in fibroblasts or bacteria were quantified with BCA method and normalized at equal amounts for the tests. The fibroblasts or bacteria in PBS were centrifuged and pelleted. The pellets were resuspended in an extraction solvent composed of ethanol: dimethyl sulfoxide: acetic acid at a ratio of 80:20:1 (vol: vol: vol) and stored at −80 °C for another 48 h. The extracts were centrifuged, and the supernatants were collected and filtered through a Sep-Pak C18 Cartridge. Excitation or emission spectra of these extracts were scanned ranging from 350 to 650 nm or from 500 to 800 nm with an excitation wavelength of 405 nm, respectively, and measured by a FLS1000 Photoluminescence Spectrometer (Edinburgh Instruments). PPIX solution at 10 μM served as a positive control. In addition, excitation and emission spectra of thymol, TQ, and THQ at 0.5 mg/mL were determined similarly.

### UPLC-VION-IMS-QTOF-MS/MS analyses of thymol photo-oxidation in viable bacterial cells and fibroblasts or their extracts

Bacterial suspension at 5 × 10^7^ CFU/mL or fibroblasts at 5 × 10^5^ cells/mL in PBS was added to 35-mm Petri dishes, along with thymol at a final concentration of 0.5 mg/mL. Sample of 100 μL was taken and centrifuged and the supernatants were collected and analyzed by UPLC-VION-IMSQTOF-MS/MS. To determine thymol photo-oxidation, the samples were irradiated with 50 J/cm^2^ BL. The BL-irradiated sample was collected and analyzed with UPLC-VION-IMSQTOF-MS/MS. In addition, the extracts prepared above were freeze-dried using a SC250 concentrator (Thermo Fisher). The resultant powder was re-dissolved in 100 μL of absolute ethanol, diluted 20x in PBS, and then supplemented with thymol solution at a final concentration of 0.5 mg/mL. The sample was taken about 100 μL from each extract for UPLC-VION-IMSQTOF-MS/MS analyses. The above extracts were irradiated with 50 J/cm^2^ BL. After BL irradiation, the sample was collected and analyzed with UPLC-VION-IMS-QTOF-MS/MS.

UPLC was performed with an ACQUITY UPLC I-class system (Waters), equipped with a binary solvent delivery system, autosampler, and a PDA detector. The separation was achieved on a Waters ACQUITY UPLC BEH C18 column (100 × 2.1 mm, 1.7 µm). The mobile phases consisted of (A) water containing 0.1% (v/v) formic acid and (B) acetonitrile containing 0.1% (v/v) formic acid. The elution condition was optimized as follows: 0–2 min, 5% B; 2–5 min, linear gradient from 5 to 25% B; 5–7 min, 50% B; 7–9 min, 50–100% B, 9–11 min, 100%, then restoration of initial conditions for 3 min to equilibrate the column. The flow rate was 0.4 mL/min. The injection sample volume was 1 µL. Mass spectrometry analysis was performed on a Vion IMS QTOF MS (Waters) equipped with an atmospheric pressure chemical ionization (APCI) source operating in a positive ionization mode. The capillary voltage and cone voltage were set at 1500 V and 40 V, respectively. The source temperature was 115 °C. The QTOF acquisition rate was 0.2 s and the inter-scan delay was 0.1 s. The mass range was scanned from 50 to 1000 *m*/*z*. The data were collected and acquired in UNIFI Scientific Information System software (Waters).

### Statistical analyses

Data are presented as means ± standard deviations (SDs). Statistical significance was assessed with two-tailed Student’s *t*-test between two groups or one-way ANOVA for multiple group comparisons. The Kaplan–Meier method was applied in the comparison of the survival curves. A *P*-value < 0.05 was considered statistically significant. All statistical analyses were performed using GraphPad Prism 7.0 (GraphPad Software).

### Reporting summary

Further information on research design is available in the Nature Research Reporting Summary linked to this article.

## Supplementary information

Supplementary Information

Description of Additional Supplementary Files

Supplementary Data

Reporting Summary

## Data Availability

All relevant data are available in this article and its Supplementary information files, except for original image files, which are available from the corresponding author upon reasonable request. All data underlying the graphs is available as Supplementary Data.
