# Supplementary material for: Bacteria-specific pro-photosensitizer kills multidrug-resistant Staphylococcus aureus and Pseudomonas aeruginosa
Source: Commun Biol. 2021 Mar 25;4:408. doi: 10.1038/s42003-021-01956-y (PMC7994569; doi:10.1038/s42003-021-01956-y)
Supplement: Supplementary file 1 — Supplementary Information [file 42003_2021_1956_MOESM1_ESM.pdf]

Supplemental Information

Bacteria-specific pro-photosensitizer kills multidrug-resistant

Staphylococcus aureus* and *Pseudomonas aeruginosa

Min Lu[1], Yongli Li[2], and Mei X. Wu[2*]

[1] Department of Orthopaedics, Shanghai Key Laboratory for Prevention and Treatment of Bone and Joint Diseases, Shanghai Institute of Traumatology and Orthopaedics, Ruijin Hospital, Shanghai Jiao Tong University School of Medicine, Shanghai 200025, P. R. China

[2] Wellman Center for Photomedicine, Massachusetts General Hospital, Department of Dermatology, Harvard Medical School, 50 Blossom Street, Boston, MA, 02114, USA

*Correspondence to Mei X. Wu (mwu5@mgh.harvard.edu)

Extended Methods

Minimum inhibitory concentration (MIC). MICs were determined by a standard broth microdilution assay as described [1, 2]. Briefly, various compounds were prepared at 50 mg/mL in N, N-Dimethylformamide (DMF) as a stock solution. Bacterial inoculum at 5×10^7 CFU/mL was seeded to a 96-well plate at 180 µL/well, to which 20 µL of BHI broth containing serial dilutions of an indicated compound was added. The medium containing a similar amount of DMF served as controls. The microplates were incubated at 37 °C for 24 hrs and the lowest concentration of the compound capable of completely inhibiting bacterial growth was referred to a MIC.

Assessment of resistance to BL combined with thymol or antibiotics alone. Possible resistance of MRSA HS0182 and *Pa* HS0028 to the combined treatment was assessed following a published protocol with some modifications [3]. Briefly, a stationary growth-phase culture at 5×10^7 CFU/mL was treated with a sublethal dose of the duo that could inhibit 3-log CFU/mL of bacterial growth. In the first inhibitory-growth cycle, the sublethal doses were 30 J/cm^2 BL plus 0.075 mg/mL thymol for MRSA HS0182 or 30 J/cm^2 BL paired with 0.15 mg/mL thymol for *Pa* HS0028. The survival bacteria were collected after the first inhibitory-growth cycle and re-cultured for the second inhibitory-growth cycle in the presence of a sublethal dose of the duo that was justified again after the first inhibitory-growth cycle. The same procedure was repeated for 20 successive cycles. Resistance was defined by any significant CFU increase of successive passages.

For comparison, resistance of MRSA HS0182 and *Pa* HS0028 to penicillin or ampicillin, respectively, was also assessed as described [4, 5]. Briefly, 180 µL of bacterial inoculum were

mixed with 20 µL of serial dilutions of antibiotics in BHI medium. After incubation at 37 °C for 24 hrs, the lowest antibiotic concentration able to completely inhibit bacterial growth was defined as a MIC. Thereafter, 10 µL of suspension grown in BHI broth containing antibiotic at 1/2 MIC was added to a fresh BHI broth. The mixtures were incubated and determined for its MIC as above. Antibiotic resistance was evaluated by any significant increases in the MIC for up to 20 passages.

Bacterial membrane damage. The bacterial membrane damage induced by the combination of BL with thymol was examined using SYTO9 and PI through confocal microscopy. The bacterial cells were exposed to BL with 0.075 mg/mL thymol for 20 minutes, then washed twice and stained with PI solution. Later, the bacterial cells were washed again and re-suspended in PBS. Finally, the cells were fixed on slides by 4% paraformaldehyde in PBS, then 10 µM of SYTO9 was added on slides. Confocal microscopy was used to visualize the green/red fluorescence.

Scanning electron microscopy (SEM). A mid-logarithmic growth-phase culture of MRSA HS0182 and *Pa* HS0028 biofilms was prepared at 1×10^6 CFU/mL with a modified TSB medium. A sterile qualitative filter paper (Whatman; pore size, 11 µm) and a stainless-steel coupon (BioSurface Technologies Corporation; diameter, 1.5 cm) were immersed in 500 µL of MRSA HS0182 or *Pa* HS0028 suspension, respectively, in a 12-well plate. After 72 hrs of incubation, the biofilms were washed and exposed to a lethal dose of BL at 75 J/cm^2 combined with 0.2 mg/mL thymol for MRSA HS0182 or 0.6 mg/mL thymol for *Pa* HS0028. The control and treated bacteria were fixed at 4 °C for 24 hrs in 0.1 M sodium cacodylate buffer containing 2.5% glutaraldehyde, 0.15% alcian blue, and 0.15% safranin O, followed by a standard

procedure of SEM sample processing. The samples were examined on a S4800 SEM (Hitachi Ltd) and micrographs were acquired under high vacuum using an accelerating voltage of 2.0 kV.

Skin toxicity study. Mice were anesthetized, shaved on the lower dorsal skin, and treated with 50 µL of thymol at 20 mg/mL in combination with 100 J/cm^2 BL as described. The treatment was applied once a day for 5 consecutive days. The skin biopsy was excised in 24 hrs after the final treatment, fixed in 10% phosphate-buffered formalin, and then embedded in paraffin. Serial tissue sections at 4 µm were cut, subject to H&E histological examination, and visualized by Nanozoomer 2.0 HT (Hamamatsu). The images were analyzed by NDP viewer software (Hamamatsu). Cell apoptosis was evaluated by the DeadEnd Fluorometric TUNEL staining per the manufacturer's instructions (Promega). Positive controls were stained in parallel using tissue sections that were pre-treated with 10 unit/mL RQ1 RNase-free DNase I to induce DNA fragmentation. Fluorescence images were captured on a FluoView FV1000-MPE confocal microscope (Olympus).

Measurements of hydrogen peroxide (H_2O_2), hydroxyl radical (•HO), and singlet oxygen ($^1O2$). H_2O_2 was measured with an Amplex Red hydrogen peroxide/peroxidase kit (Fisher Scientific) per the manufacturer's instruction [6]. In brief, thymol, TQ, and THQ were prepared at 50 mg/mL in N, N-dimethylformamide and diluted into a final concentration of 0.2 mg/mL in PBS. The compound was added to the samples, followed immediately with BL illumination at 50 J/cm^2. The treated samples were aliquoted to 50 µL, mixed with an equal amount of the Amplex Red reagent/HRP working solution, and incubated at room temperature for 20 minutes. The amount of H_2O_2 was determined by a microplate spectrophotometer (Molecular Devices) at an

excitation/emission wavelength of 571/585 nm. Similarly, •HO was measured by the molecular probe 3’-(p-hydroxyphenyl) fluorescein (HPF) in accordance with the manufacturer’s instruction (Invitrogen) [7]. In brief, the samples to be assayed were added with thymol, TQ, or THQ at 0.2 mg/mL and then seeded into a 96-well plate at 180 µL/well, preincubated with 20 µL of HPF at a final concentration of 5 µM for 15 minutes, followed by exposure to 50 J/cm^2 BL. •HO was quantified by a microplate spectrophotometer (Molecular Devices) at excitation/emission wavelength of 490/515 nm. $^1O_2$ was measured by a singlet oxygen sensor green reagent (SOSG) (Invitrogen) [8]. Briefly, $^1O_2$ levels in PPIX solution at 10 µM, MRSA HS0182 or *Pa* HS0028 suspension, or fibroblast were quantified by a microplate spectrophotometer (Molecular Devices) at excitation/emission wavelength of 490/515 nm and 505/525 nm, respectively, after indicated treatments in the presence or absence of 50 J/cm^2 BL and/or 10 µM NaN_3.

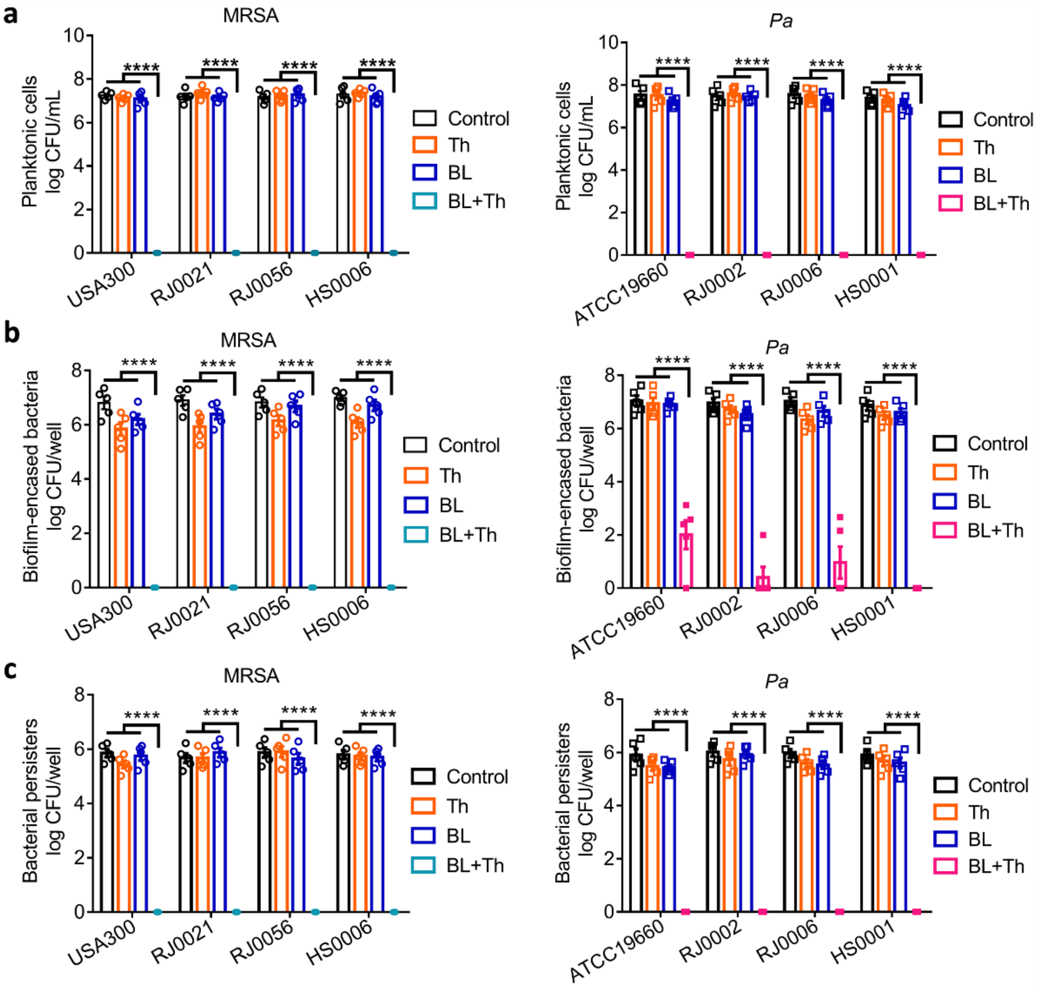

Fig. S1 Bactericidal efficacy of BL and/or thymol against planktonic cells (A), established biofilms (b), and bacterial persisters (c) of a panel of MDR MRSA and *Pa* strains.

Bactericidal efficacy of sham light (control), BL illumination at 30 J/cm^2 (BL), thymol at MIC (Th), or both (BL+Th) against MRSA (left) and *Pa* (right) planktonic cells (**a**). BL at 100 J/cm^2 combined with thymol at MIC was similarly employed to treat established biofilm (**b**) or persisters (**c**). Persisters were derived from mature biofilms of MRSA and *Pa* biofilms after treatment for 24 hrs with 100 × MICs of rifampicin or ciprofloxacin, respectively, as detailed in Materials and Methods. **** $p<0.0001$ in BL+Th groups in comparison with control and monotreatment. Results are represented as Mean ± SD of four to six replicates from three independent experiments.

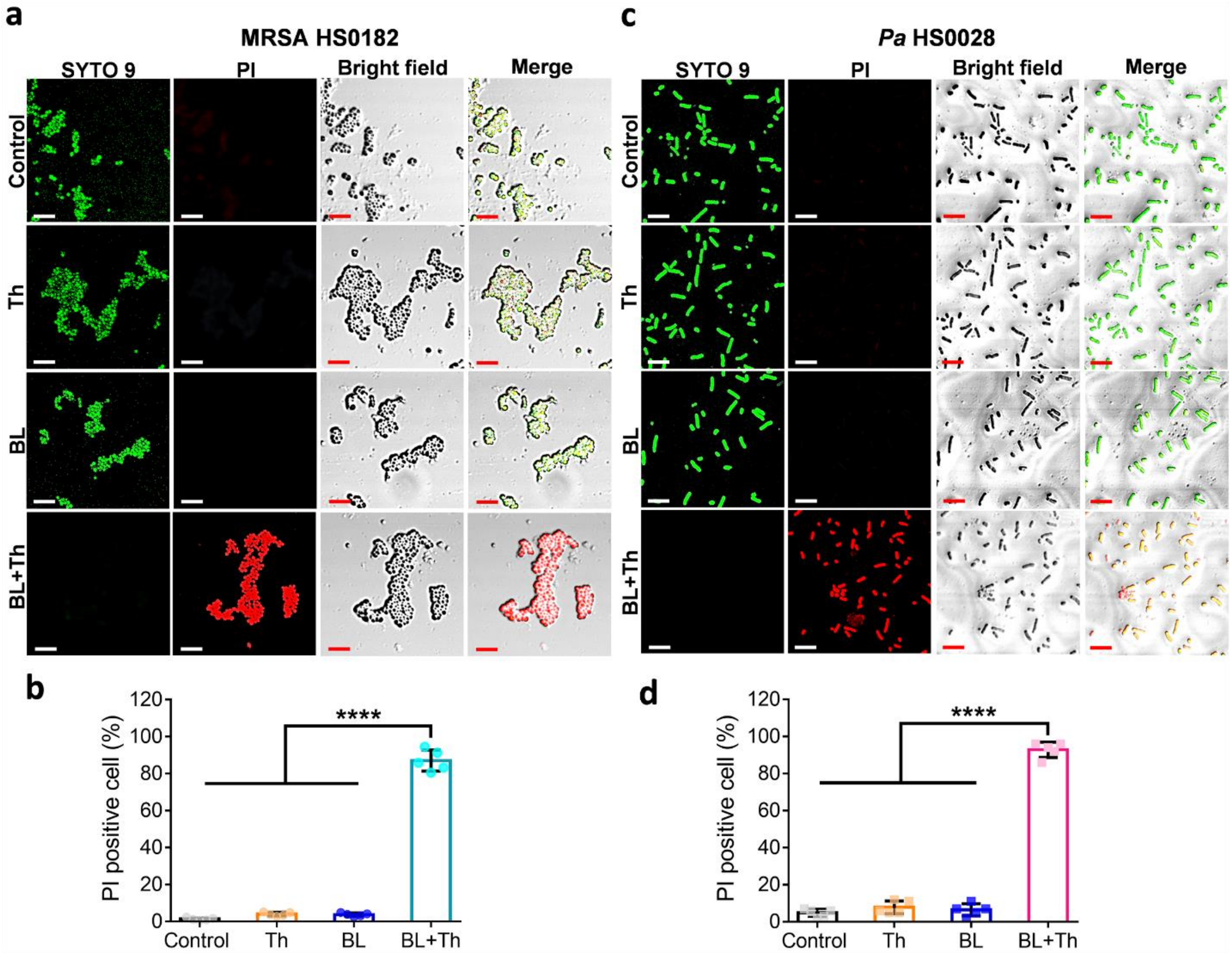

Fig. S2 Confocal microscopy of live/dead MRSA HS0182 and *Pa* HS0028 following an indicated treatment. a and **c** Representative fluorescence and phase-contrast images of MRSA HS0182 (**a**) and *Pa* HS0028 (**c**) treated with sham, thymol (0.15 mg/mL for MRSA HS0182 and 0.3 mg/mL for *Pa* HS0028), BL (50 J/cm^2 for MRSA HS0182 and 25 J/cm^2 for *Pa* HS0028), or both. The dead cells were visualized by PI staining (red), while the SYTO 9 (green) marked all live cells. Scale bar, 10 μm. All images are representative of five independent experiments. **b** and **d** PI stained MRSA HS0182 (**b**) and *Pa* HS0028 (**d**) cells were counted and expressed as percentages of total cell. **** p<0.0001 in BL+Th-treated group compared with control-, Th-, and BL-treated groups. Results are presented as Mean ± SD of five independent experiments.

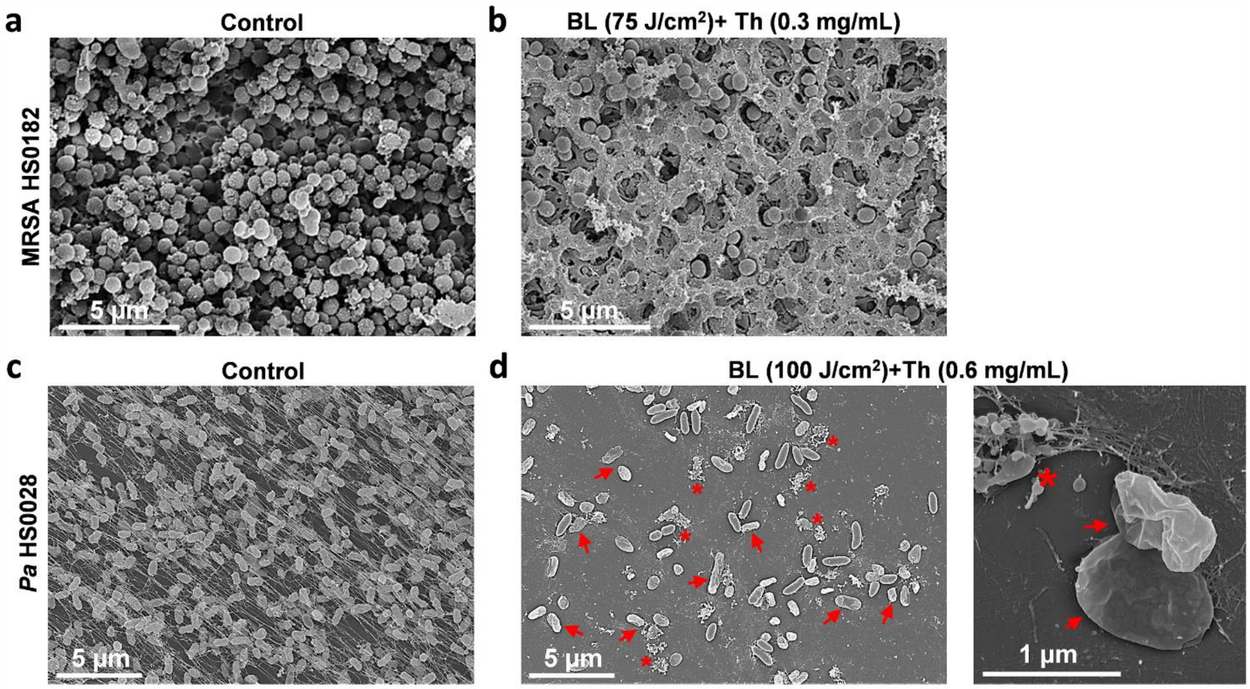

Fig. S3 Representative scanning electron micrographs of MRSA HS0182 and *Pa* HS0028 biofilms before and after an indicated treatment. MRSA HS0182 and *Pa* HS0028 biofilms were grown on sterile qualitative filter paper (Millipore; pore size, 0.22 μm) or stainless-steel coupon (BioSurface Technologies Corporation, 11 μm), respectively. After 72 hours of incubation, the formed biofilms were treated with sham (**a** and **c**) or a lethal dose of BL combined with thymol (**b** and **d**), and then imaged by scanning electron microscopy (SEM). Arrows and asterisks indicate cell collapse and cell decomposition, respectively. Images are representative of five independent experiments.

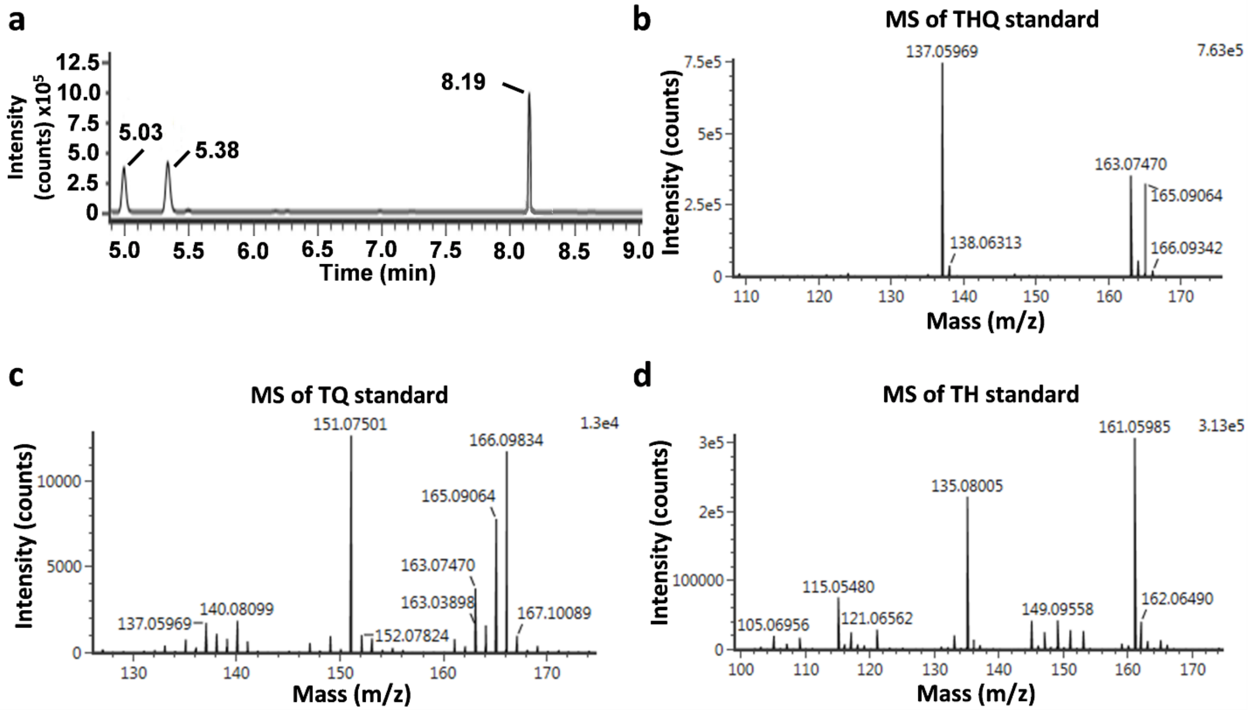

Fig. S4 UPLC-VION-IMS-QTOF-MS/MS analyses of thymol, THQ, and TQ standards. a The peak times of THQ, TQ, and thymol standards are 5.03 min, 5.38 min, and 8.19 min, respectively. The mass spectra of THQ, TQ, and thymol standards are shown in **b**, **c**, and **d**, respectively.

Table S1 MIC values of MRSA and *Pa* strains to various antibiotics. Ten bacterial strains were used in this study, including four MRSA clinical isolates (RJ0021, RJ0056, HS0006, and HS0182), four *Pa* clinical isolates (RJ0002, RJ0006, HS0001, and HS0028), one luminescent strain of USA300, and one standard strain of *Pa* ATCC19660.

	MRSA (MIC, μg/mL)					*Pa* (MIC, μg/mL)				
Antibiotics	USA300	RJ0021	RJ0056	HS0006	HS0182	ATCC 19660	RJ0002	RJ0006	HS0001	HS0028
AMK						32	>32	64	64	>32
AMP						16	>16	32	16	32
MEM						>8	8	16	>16	8
ATM						16	32	32	32	16
SXT						8	>16	16	>8	16
FEP						>2	16	>2	16	32
TGC						>16	16	32	32	32
CAZ						64	>32	128	>64	>64
CRO						>64	64	128	128	64
CXM						32	>16	>32	64	64
CIP						>16	>8	16	32	16
CLI	>8	16	16	8	>4					
DAP	1	<1	1	<1	<1					
ERY	>8	8	8	>8	16					
GEN	>16	16	32	>8	16					
IPM						16	>8	2	32	32
LVX	>8	8	16	32	64					
LNZ	16	8	>16	32	16					
LZD	>1	<1	2	1	1					
MXF	>8	8	16	8	32					
NIT						64	128	>128	128	128
OXA	16	8	32	32	16					
RIF	1	<1	2	1	2					
PEN	2	<1	<1	1	<1					
TET	>8	2	2	1	>4					
CZT						>16	32	>32	64	32
VAN	1	<1	<1	1	>4					

Antibiotics: AMK, amikacin; AMP, ampicillin; MEM, meropenem; ATM, aztreonam; SXT, trimethoprim-sulfamethoxazole; FEP, cefepime; TGC, tigecycline; CAZ, ceftazidime; CRO, ceftriaxone; CXM, cefuroxime; CIP, ciprofloxacin; CLI, clindamycin; DAP, daptomycin; ERY, erythromycin; GEN, gentamicin; IPM, imipenem; LVX, levofloxacin; LNZ, linezolid; LZD, linezolid; MXF, moxifloxacin; NIT, nitrofurantoin; OXA, oxacillin; RIF, rifampin; PEN, penicillin; TET, tetracycline; CZT, ceftizoxime; and VAN, vancomycin.

References

1. Nakatsuji T*, et al.* Antimicrobial Property of Lauric Acid Against Propionibacterium Acnes: Its Therapeutic Potential for Inflammatory Acne Vulgaris. *J Invest Dermatol* **129**, 2480-2488 (2009).

2. Pablos C*, et al.* Novel antimicrobial agents as alternative to chlorine with potential applications in the fruit and vegetable processing industry. *Int J Food Microbiol* **285**, 92-97 (2018).

3. Zhang Y*, et al.* Antimicrobial blue light therapy for multidrug-resistant Acinetobacter baumannii infection in a mouse burn model: Implications for prophylaxis and treatment of combat-related wound infections. *J Infect Dis* **209**, 1963-1971 (2014).

4. de Breij A*, et al.* The antimicrobial peptide SAAP-148 combats drug-resistant bacteria and biofilms. *Sci Transl Med* **10**, (2018).

5. Li X*, et al.* Functional Gold Nanoparticles as Potent Antimicrobial Agents against Multi-Drug-Resistant Bacteria. *ACS Nano* **8**, 10682-10686 (2014).

6. Jiang Z, Woollard ACS, Wolff SP. Hydrogen peroxide production during experimental protein glycation. *FEBS lett* **268**, 69-71 (1990).

7. Morones-Ramirez JR, Winkler JA, Spina CS, Collins JJ. Silver enhances antibiotic activity against gram-negative bacteria. *Sci Transl Med* **5**, 1-12 (2013).

8. Huang L, Xuan W, Zadlo A, Kozinska A, Sarna T, Hamblin MR. Antimicrobial photodynamic inactivation is potentiated by the addition of selenocyanate: Possible involvement of selenocyanogen? *J Biophotonics* **11**, 1-10 (2018).
