# Supplementary material for: Bacteria-specific pro-photosensitizer kills multidrug-resistant Staphylococcus aureus and Pseudomonas aeruginosa
Source: Commun Biol. 2021 Mar 25;4:408. doi: 10.1038/s42003-021-01956-y (PMC7994569; doi:10.1038/s42003-021-01956-y)
Supplement: Supplementary file 2 — Description of Additional Supplementary Files [file 42003_2021_1956_MOESM2_ESM.pdf]

Description of Additional Supplementary Files

File name: Supplementary Data

Description: Raw data underlying Fig. 1b, Fig. 1c, Fig. 2, Fig. 3b-3i, Fig. 4b-4g, 5a5c, Fig. 5e, Fig. 6d, Fig. 6g, and Fig. 6h.
