# Supplementary material for: Bacteria-specific pro-photosensitizer kills multidrug-resistant Staphylococcus aureus and Pseudomonas aeruginosa
Source: Commun Biol. 2021 Mar 25;4:408. doi: 10.1038/s42003-021-01956-y (PMC7994569; doi:10.1038/s42003-021-01956-y)
Supplement: Supplementary file 4 — Reporting Summary [file 42003_2021_1956_MOESM4_ESM.pdf]

nature research

Corresponding author(s): Mei X. Wu

Last updated by author(s): Feb 18, 2021

Reporting Summary

Nature Research wishes to improve the reproducibility of the work that we publish. This form provides structure for consistency and transparency in reporting. For further information on Nature Research policies, see our Editorial Policies and the Editorial Policy Checklist.

Statistics

For all statistical analyses, confirm that the following items are present in the figure legend, table legend, main text, or Methods section.

n/a	Confirmed	
☐	☒	The exact sample size (*n*) for each experimental group/condition, given as a discrete number and unit of measurement
☐	☒	A statement on whether measurements were taken from distinct samples or whether the same sample was measured repeatedly
☐	☒	The statistical test(s) used AND whether they are one- or two-sided<br>*Only common tests should be described solely by name; describe more complex techniques in the Methods section.*
☐	☒	A description of all covariates tested
☐	☒	A description of any assumptions or corrections, such as tests of normality and adjustment for multiple comparisons
☐	☒	A full description of the statistical parameters including central tendency (e.g. means) or other basic estimates (e.g. regression coefficient) AND variation (e.g. standard deviation) or associated estimates of uncertainty (e.g. confidence intervals)
☐	☒	For null hypothesis testing, the test statistic (e.g. *F*, *t*, *r*) with confidence intervals, effect sizes, degrees of freedom and *P* value noted<br>*Give P values as exact values whenever suitable.*
☒	☐	For Bayesian analysis, information on the choice of priors and Markov chain Monte Carlo settings
☒	☐	For hierarchical and complex designs, identification of the appropriate level for tests and full reporting of outcomes
☒	☐	Estimates of effect sizes (e.g. Cohen's *d*, Pearson's *r*), indicating how they were calculated

Our web collection on statistics for biologists contains articles on many of the points above.

Software and code

Policy information about availability of computer code

Data collection	No custom software was used
Data analysis	Excel (office 365), GraphPad Prism 7.0 (GraphPad Software), IVIS imaging system, FlowJo_V10

For manuscripts utilizing custom algorithms or software that are central to the research but not yet described in published literature, software must be made available to editors and reviewers. We strongly encourage code deposition in a community repository (e.g. GitHub). See the Nature Research guidelines for submitting code & software for further information.

Data

Policy information about availability of data

All manuscripts must include a data availability statement. This statement should provide the following information, where applicable:

- Accession codes, unique identifiers, or web links for publicly available datasets
- A list of figures that have associated raw data
- A description of any restrictions on data availability

All relevant data are available in this article and its Supplementary information files, except for original image files, which are available from the corresponding author upon reasonable request. All raw datas underlying underlying Fig. 1b, Fig. 1c, Fig. 2, Fig. 3b-3i, Fig. 4b-4d, Fig. 4e-4g, 5a-5c, Fig. 5e, Fig. 6d, Fig. 6g, and Fig. 6h are available as Supplementary Data

Field-specific reporting

Please select the one below that is the best fit for your research. If you are not sure, read the appropriate sections before making your selection.

☒ Life sciences ☐ Behavioural & social sciences ☐ Ecological, evolutionary & environmental sciences

For a reference copy of the document with all sections, see nature.com/documents/nr-reporting-summary-flat.pdf

Life sciences study design

All studies must disclose on these points even when the disclosure is negative.

Sample size	All sample size are determined based on the statistically difference analysis. All sample size is indicated for each experiment in the corresponding figure legend
Data exclusions	All data are shown in the corresponding figures in our manuscript
Replication	All results are presented as Mean±SD of at least five independent experiments. All images are representative of five independent experiments. The details are shown in corresponding figure legend in our manuscript
Randomization	The BALB/C mice used in this study are allocated randomly
Blinding	Yes, all experiments are blinded to group allocation during data collection and/or analysis

Reporting for specific materials, systems and methods

We require information from authors about some types of materials, experimental systems and methods used in many studies. Here, indicate whether each material, system or method listed is relevant to your study. If you are not sure if a list item applies to your research, read the appropriate section before selecting a response.

Materials & experimental systems

n/a	Involved in the study	
☒	☐	Antibodies
☐	☒	Eukaryotic cell lines
☒	☐	Palaeontology and archaeology
☐	☒	Animals and other organisms
☒	☐	Human research participants
☒	☐	Clinical data
☒	☐	Dual use research of concern

Methods

n/a	Involved in the study	
☒	☐	ChIP-seq
☐	☒	Flow cytometry
☒	☐	MRI-based neuroimaging

Eukaryotic cell lines

Policy information about cell lines

Cell line source(s)	The human fibroblasts were purchased from ATCC (ATCC PCS-201-012)
Authentication	The cell line is authenticated by ATCC
Mycoplasma contamination	The cell line tested nagative for mycoplasma contamination based on the information provided by ATCC
Commonly misidentified lines (See ICLAC register)	n/a

Animals and other organisms

Policy information about studies involving animals; ARRIVE guidelines recommended for reporting animal research

Laboratory animals	BALB/C mice, female, at 8 weeks of age
Wild animals	n/a
Field-collected samples	n/a
Ethics oversight	All animal protocols were approved by the Shanghai Jiao Tong University Animal Study Committee

Note that full information on the approval of the study protocol must also be provided in the manuscript.

Flow Cytometry

Plots

Confirm that:

- [x] The axis labels state the marker and fluorochrome used (e.g. CD4-FITC).
- [x] The axis scales are clearly visible. Include numbers along axes only for bottom left plot of group (a 'group' is an analysis of identical markers).
- [x] All plots are contour plots with outliers or pseudocolor plots.
- [x] A numerical value for number of cells or percentage (with statistics) is provided.

Methodology

Sample preparation	Please find these descriptons in materials and methods section
Instrument	Flow cytometry (BD Biosciences)
Software	FlowJo_v10
Cell population abundance	n/a
Gating strategy	The gates are set up using FlowJo_v10 software based on unstained sample and control samples

- [x] Tick this box to confirm that a figure exemplifying the gating strategy is provided in the Supplementary Information.
